# Actomyosin based contraction: one mechanokinetic model from single molecules to muscle?

**DOI:** 10.1007/s10974-016-9458-0

**Published:** 2016-11-18

**Authors:** Alf Månsson

**Affiliations:** Department of Chemistry and Biomedical Sciences, Faculty of Health and Life Sciences, Linnaeus University, 39182 Kalmar, Sweden

**Keywords:** Striated muscle, Single molecules, In vitro motility assay, Myosin, Actin, Monte-Carlo simulation

## Abstract

Bridging the gaps between experimental systems on different hierarchical scales is needed to overcome remaining challenges in the understanding of muscle contraction. Here, a mathematical model with well-characterized structural and biochemical actomyosin states is developed to that end. We hypothesize that this model accounts for generation of force and motion from single motor molecules to the large ensembles of muscle. In partial support of this idea, a wide range of contractile phenomena are reproduced without the need to invoke cooperative interactions or ad hoc states/transitions. However, remaining limitations exist, associated with ambiguities in available data for model definition e.g.: (1) the affinity of weakly bound cross-bridges, (2) the characteristics of the cross-bridge elasticity and (3) the exact mechanistic relationship between the force-generating transition and phosphate release in the actomyosin ATPase. Further, the simulated number of attached myosin heads in the in vitro motility assay differs several-fold from duty ratios, (fraction of strongly attached ATPase cycle times) derived in standard analysis. After addressing the mentioned issues the model should be useful in fundamental studies, for engineering of myosin motors as well as for studies of muscle disease and drug development.

**Electronic supplementary material:**

The online version of this article (doi:10.1007/s10974-016-9458-0) contains supplementary material, which is available to authorized users.

## Introduction

It is well-known that contraction of striated muscle (skeletal muscle and heart) results from interactions, driven by ATP-turnover, between myosin and actin (Månsson et al. 2015). The arrangement of these proteins in large ordered ensembles, sarcomeres, in striated muscle ensures highly optimized function as indicated by convergent evolution in organisms as distant as cnidarians (e.g. jellyfish) and mammals (Steinmetz et al. 2012). Despite intense studies, the basis for the high level of perfection of the ordered system remains poorly understood (Batters et al. 2014; Månsson et al. 2015). This fact is not only of fundamental importance. Increased insight in this regard would also benefit the understanding and treatment of diseases, e.g. cardiomyopathies (Ferrantini et al. 2009; Frey et al. 2012; Green et al. 2016; Marston 2011; Spudich 2014; Teekakirikul et al. 2012), a leading cause of sudden death in young people, and heart failure, a leading cause of hospitalization and mortality where myosin-active drugs may be useful (Malik et al. 2011; Teerlink et al. 2011). One reason for remaining knowledge gaps is the difficulty to integrate experimental results as well as key concepts, from studies using different techniques and hierarchical scales ranging from single molecules over small ensembles to muscle (Baker et al. 2002; Månsson et al. 2015; Walcott et al. 2012). One tool that would be valuable in this regard is a mathematical model that bridges the gap while being both simple enough to effectively aid data interpretation and sufficiently detailed to incorporate biochemical actomyosin states that are critical for function.

The in vitro motility assay (Kron and Spudich 1986) is a powerful small ensemble assay that is increasingly used for studies of muscle disorders with potential usefulness in drug development (Aksel et al. 2015; Kohler et al. 2003; Li and Larsson 2010; Sommese et al. 2013; Song et al. 2011). Generally, fluorescence-labeled actin filaments are propelled by surface adsorbed myosin molecules or myosin motor fragments obtained by mild proteolysis or expression in cell systems (Kron et al. 1991). The assay may be combined with genetic engineering (Kubalek et al. 1992; Schindler et al. 2014; Sommese et al. 2013; Tsiavaliaris et al. 2004) and has been adapted for single molecule studies e.g. using optical tweezers (Finer et al. 1994), nm tracking techniques (Wang et al. 2013) or cantilever systems (Ishijima et al. 1996; Kalganov et al. 2013) to measure pN–nN forces and nm displacements.

Mathematical models that cover the scale from single molecules to large ensembles are required to relate results of the in vitro assays to molecular properties on the one hand and muscle contractile function in health and disease on the other. Currently, however, key data obtained from small ensembles and the large ensembles of muscle are generally interpreted using different theoretical frameworks. For instance, elasticity is central in muscle function and, therefore, also in classical models (Hill 1974; Huxley 1957; Huxley and Simmons 1971) of muscle contraction where changes in state of one head or a group of heads are assumed to influence the strain, and thereby strain-dependent kinetics, of other heads. In contrast, certain in vitro motility assay data are interpreted using models (Uyeda et al. 1990; Wang et al. 2013) without such elastic coupling between motors. Furthermore, the precise geometrical arrangement of the myofilament lattice in muscle is absent in the different versions of the in vitro motility assay. On the other hand, this arrangement is implicit in most models of muscle contraction (Eisenberg and Hill 1978; Huxley 1957; Månsson 2010) since the ensemble-averaged forces, turnover rates etc. that are considered in these models rely on the actin-myosin organization of the muscle sarcomere.

Efforts have been made to include elastic coupling between cross-bridges in models for actomyosin behavior in the vitro motility assay (Baker et al. 2002; Erdmann and Schwarz 2012; Hilbert et al. 2013; Ishigure and Nitta 2015; Pate and Cooke 1991; Walcott et al. 2012). Studies have also addressed the role of the hierarchical organization using spatially explicit models of large ensembles of actin and myosin (Campbell 2009; Tanner et al. 2007) with aims to elucidate the roles of the geometrical arrangement in specific situations. However, these models (Baker et al. 2002; Campbell 2009; Erdmann and Schwarz 2012; Hilbert et al. 2013; Ishigure and Nitta 2015; Pate and Cooke 1991; Tanner et al. 2007; Walcott et al. 2012) have used simplified representations of the actin-myosin ATP turnover mechanism. For instance, like several previous large-ensemble muscle models (e.g. (Duke 1999; Eisenberg et al. 1980; Månsson 2010; Pate and Cooke 1989; Persson et al. 2013), the weakly bound actomyosin state has not been explicitly included. Neither has the phosphate release step been separated from the main force-generating event (“the power-stroke”). Further, the full scale from single molecules to the large ensembles of muscle has not been considered. All this makes it difficult to use the models for elucidating e.g. disease mechanisms (see above) or drug effects that involve stabilization of a specific biochemical state suggested by solution kinetics studies.

With the aim to overcome current limitations, we here investigate to what extent a statistical actomyosin cross-bridge model of classical type (Hill 1974; Huxley 1957) with detailed biochemical cycle but without explicit geometrical details can account for contractile behavior from single molecules to large ensembles of muscle. Due to the ample evidence for cooperativity or emergent phenomena during active lengthening and under conditions of varied activation (Brunello et al. 2007; Edman and Flitney 1982; Gordon et al. 2000; Linari et al. 2015; McKillop and Geeves 1993; Rassier 2012b) the studies focus on cases of full activation and isometric contraction or shortening contraction. However, it is briefly considered how activation mechanisms may be introduced as well as certain mechanisms that are specific to lengthening contractions. The model is defined on basis of kinetic parameter values from the literature obtained under as coherent conditions as possible (with regard to temperature, ionic strength, muscle type etc.).

As a basis for the investigations, it is hypothesized that such a coherently defined actomyosin model accounts for generation of force and motion from single molecules to the ordered arrays in muscle without the need to invoke emergent phenomena and/or consider structural details. This hypothesis was largely supported as it was found that the model, surprisingly well, approximates key contractile phenomena on different scales. However, there were remaining limitations in details of model performance. These limitations provide mechanistic insights as discussed in detail below. They also suggest improvements in the details of the model as well as new experimental studies. After such further developments the model should be useful in studies of muscle diseases and in guiding associated drug development. We also consider possible usefulness in guiding engineering of myosin motors (Amrute-Nayak et al. 2010; Nakamura et al. 2014; Schindler et al. 2014; Tsiavaliaris et al. 2004) e.g. for optimized use in nanotechnological applications (Kumar et al. 2016; Månsson 2012; Nicolau et al. 2016).

## Materials and Methods

For large ensembles of myosin motors, the model was implemented by solving differential equations in state probabilities using Simnon software (version 1.3; SSPA, Gothenburg, Sweden). For small ensembles and single molecules, the model was implemented using a Monte-Carlo approach (Gillespie 1976). Simulation details are given in the Supporting Information.

In vitro motility assay velocities (v_f_) as function of the number (N) of available myosin head were fitted to the equation (Uyeda et al. 1990):

1 $$v_{f} = v^{\infty } (1 - \left( {1 - f} \right)^{N} ) \, = v^{\infty } (1 - \left( {1 - f} \right)^{\rho dl} )$$

Here, *v* ^*∞*^ is the sliding velocity for infinitely long filaments, f is a myosin duty ratio (fraction of ATP-turnover time spent in strongly bound states). The quantity N = *ρdl,* where l is filament length, *ρ* is the surface density of active myosin heads and *d* is the width of band around the actin filament where myosin heads reach their attachment sites on the filament. Previously we found, using trimethylchlorosilane-coated surfaces, that ρ ≈ 5000 µm^−2^ and d ≈ 30 nm. Equations were fitted to simulated data using non-linear regression (Marquardt–Levenberg algorithm; GraphPad Prism v. 6.05).

### Theory

**Fig. 1:**
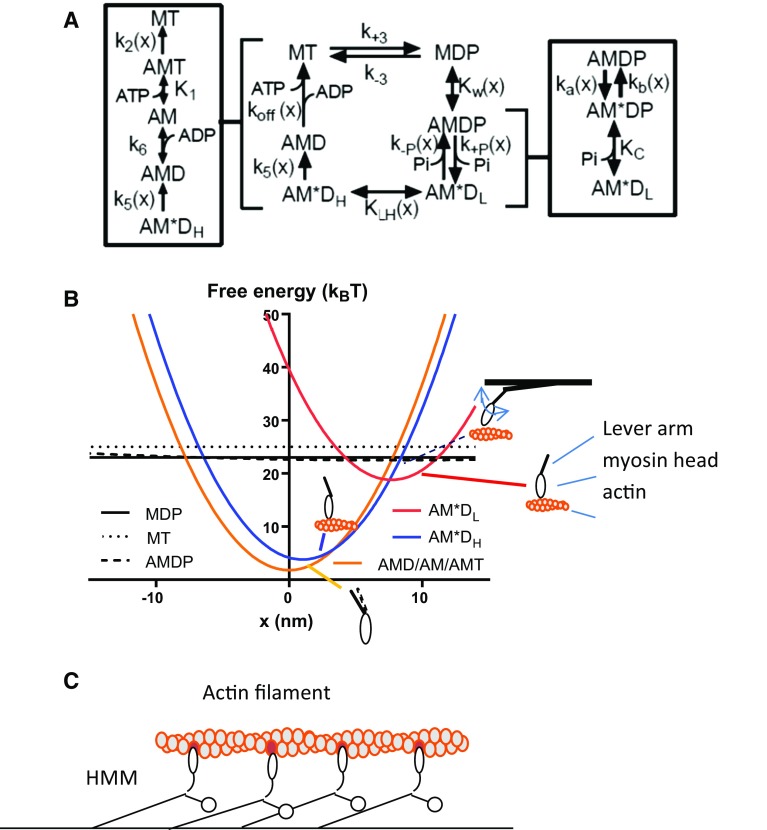
Key elements of model. **a** Kinetic scheme showing myosin (M) and actomyosin (AM) biochemical states with MgATP (T), MgADP (D) and/or inorganic phosphate (P/Pi) at the active site. The MDP and AMDP states, on the one hand, and the AM*D_L_ and AM*D_H_ states, on the other, are assumed to be in rapid equilibrium with equilibrium constants K_w_(x) and K_LH_(x), respectively. The AM*D_L_ state is a strongly bound, start-of-power-stroke state. Several rate constants k_i_ as well as equilibrium constants K_i_ vary with the elastic strain in the cross-bridge. **b** The free energy of all states as function of x (see text). Insets schematically illustrate different structural states of myosin head and lever arm at free-energy minima. **c** Schematic illustration of an in vitro motility assay where myosin motor fragments (heavy meromyosin; HMM) are adsorbed to a surface. The one-head, single-site assumption is illustrated: only one of the two myosin heads can bind to actin and binding is possible only to one site (*dark*) per 36 nm helical half-repeat of the actin filament. Key parameter values are given in Supplementary Tables S1 and S2

The model is based on the kinetic scheme in Fig. 1a with abbreviations of different states explained in the legend. The total amplitude of the force-generating power-stroke (“step-length”; from state AM*D_L_ to states AMD and AM) was taken as 7.7 nm, slightly modified from the value of 7.35 nm in a recent model for large ensembles (Persson et al. 2013).The value of 7.7 nm is consistent with an 8 nm myosin step suggested in (Kaya and Higuchi 2010) based on optical tweezers studies that did not distinguish sub-steps. By assuming a final sub-step of 1 nm (Capitanio et al. 2006) the total value of 7.7 nm suggests a first sub-step of 6.7 nm. These step-lengths are directly reflected in the diagrams in Fig. 1b that illustrate the free energy of different states as function of the variable x. This variable represents the distance between a reference point on the myosin molecule and the nearest actin filament site, with x = 0 nm when force in the AMD/AM state is zero. The free energies of two different generic states, i and j, are related to the equilibrium constant (K_ij_(x)) and forward and backward rate constants (k_ij_(x) and k_ji_(x)) as follows:

2 $${\text{K}}_{\text{ij}} \left( {\text{x}} \right) = {\text{k}}_{\text{ij}} \left( {\text{x}} \right)/{\text{k}}_{\text{ji}} \left( {\text{x}} \right) = \exp \left( {\left( {{\text{G}}_{\text{i}} \left( {\text{x}} \right) - {\text{G}}_{\text{j}} \left( {\text{x}} \right)} \right)/{\text{k}}_{\text{B}} {\text{T}}} \right)$$

All rate functions used are defined in detail the Supplementary Theory and related parameter values are given in Supplementary Tables S1 and S2. We did not explicitly include an intermediate AM*DP-state (Dantzig et al. 1992) (between the AMDP and the AM*D_L_ states) in the free energy diagrams in Fig. 1b. However, such a state is implicit in the strain-dependence of the rate constant for the weak-to-strong (AMDP to AM*D_L_) transition (Supplementary Eq. S2) and the reversal of this transition (Supplementary Eqs. S3 and S4). The strain-dependence of free energy of the implicit AM*DP state is assumed to be identical to that for the AM*D_L_ state (Fig. 1b), but displaced upwards by a quantity k_B_T ln(K_C_/[Pi]).

The MgATP-induced detachment (k_2_(x)) was generally taken as strain-dependent, an issue that is discussed below (see also Supplementary Theory).

The effect of including a fraction of non-cycling “dead” myosin heads (e.g. treated with *N*-ethyl maleimide; NEM) in the in vitro motility assay (e.g. Aksel et al. 2015; Bing et al. 2000) was simulated by introducing two additional states in the model, one attached to actin (AM^D^; “^D”^ for “dead”) and one detached (M^D^). The detachment kinetics was taken from optical tweezer results for HMM-actin in rigor (Nishizaka et al. 2000). The M^D^ and AM^D^ states were assumed to have free-energy diagrams similar to that of the MT (not varying with x) and AM state, respectively with a difference of 18 k_B_T (unless otherwise stated) between the free energy minima of the states. The kinetic scheme governing the transitions between the AM^D^ and M^D^ states was assumed separated from the scheme in Fig. 1a. That is, the only allowed transitions for non-cycling heads were between the M^D^ and AM^D^ states. For further details, see Supplementary Methods.

The present model assumes maximum Ca^2+^ activation as defined in detail in the Supplementary Theory. It is also assumed (Fig. 1c) that a maximum of one site on the actin filament is within reach of any myosin cross-bridge and that the thin/actin and thick/myosin filaments are infinitely stiff. Furthermore, only one of the two globular units (heads) of each myosin molecule is assumed to bind simultaneously to actin. The neighboring sites on the actin filament are generally assumed to be separated by 36 nm but in some control simulations, we consider the effects of assuming three independent sites per 36 nm.

All strongly bound myosin heads are assumed to have a stiffness of 2.8 pN/nm whereas the stiffness of weakly bound myosin heads is taken as 0.02 pN/nm. This is consistent with the weak non-specific attachment and appreciable rotational freedom of the latter type of heads. In most cases, we assume similar stiffness for positive and negative x but we also consider non-linear elasticity of strongly bound heads with cross-bridge stiffness, 0.25 pN/nm for x < 0 nm (see Supplementary Theory).

The model is defined on basis of kinetic parameter values from the literature, obtained under as coherent conditions as possible (with regard to temperature [~30 °C]), ionic strength [130–200 mM], muscle type [fast rabbit skeletal muscle] etc.). In the case of deviations from the pre-selected conditions we either make corrections, when possible, or discuss the consequences of the deviations (Supplementary Tables S1 and S2 and Supplementary Theory).

## Results

Simulation of muscle contractile properties gave similar results whether using numerical solution of differential equations or Monte-Carlo simulations (Supplementary Fig. S1). This suggests that the two approaches can be used interchangeably for large ensembles. Unless otherwise stated, the parameter values in Supplementary Tables S1 and S2 were used and [MgATP] = 5 mM. The value used for k_5_ (Fig. 1a) required particular considerations as discussed below and in Supporting Theory.

Using standard parameter values, the kinetic scheme in Fig. 1a predicts that Vmax ≈ 89 s^−1^ for actomyosin ATPase in solution at saturating [actin] (Supplementary Eq. S14). This is similar to 86 s^−1^ in experiments (temperature correction from (Brenner and Eisenberg 1986) assuming Q_10_ = 2.7). With an effective actin concentration of 1–3 mM (Brenner et al. 1986), a binding constant K_w_ = 1 corresponds to a binding constant K_a_ in solution in the range 0.4–1.2 × 10^3^ M^−1^ and K_M_ in the range 0.4–1.2 mM for the actomyosin ATPase (actin concentration for half-maximum ATPase) (Supplementary Eq. S15). This is similar to experimental results (Woledge et al. 1985). On the other hand, if K_w_ is an order of magnitude higher (e.g. K_w_ = 12), then K_a_ would be overestimated in the model, being in the range 4–12 × 10^3^ M^−1^ with K_M_ ≈ 0.04–0.12 mM. However, as pointed out below, if we assume K_w_ = 12, this gives better fit of the model to the force–velocity relationship and other mechanics data. Whereas K_w_ = 12 leads to overestimation of K_a_ if the effective actin concentration is 1–3 mM it is important to note that there is some uncertainty in the latter value (see “Discussion” section).

**Fig. 2:**
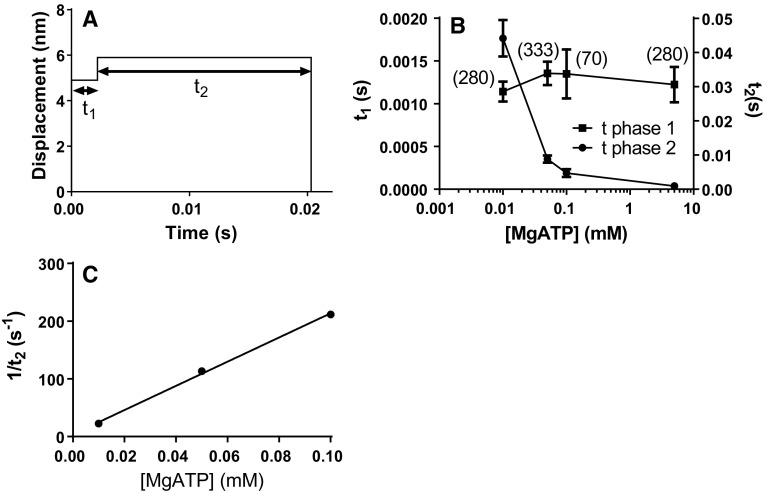
Predictions of single molecule mechanics. **a** Typical simulated displacement record following attachment of single myosin molecule to actin filament at zero time. Brownian noise not simulated. **b** [MgATP] dependence of the average duration of first (t_1_) and second (t_2_) phases of displacement records like in A. *Error bars* SEM. Number of simulated events given in parentheses. **c** The rate constant 1/t_2_ with (t_2_ from B without *error bars*) plotted vs [MgATP] at [MgATP]≤ 0. 1 mM

The model predicts two length steps in single molecule recordings (Fig. 2; cf. Capitanio et al. 2006) with average amplitudes of 6.55 ± 3.33 nm (standard deviation; n = 994) and 1 nm, respectively. Here, the first step varies in amplitude due to a spectrum of x-positions for initial cross-bridge attachment. The results are consistent with total amplitude of ~8 nm at low optical trap stiffness (Kaya and Higuchi 2010). In terms of the model, the 6.6 nm step directly corresponds to the difference in x-position of the free-energy minima for the AM*D_L_ and the AM*D_H_ states (Fig. 1b) whereas the 1 nm step corresponds to the x-difference between the minimum free energy of the AM*D_H_ and the AMD/AM states. This clearly illustrates the correspondence between single molecule length-step data and free-energy diagrams of the Hill type (Hill 1974).

The two length-steps separate the tension response into two phases (Fig. 2a). The duration of the first phase (t_1_) is independent of [MgATP] whereas the second phase exhibits [MgATP]-dependent duration (t_2_; Fig. 2b) with rate constant proportional to [MgATP] at low [MgATP] (Fig. 2c; cf. Capitanio et al. 2006).

The load-dependence of the ATP induced detachment rates in single molecule recordings shows similarities to experiments (Capitanio et al. 2012; Sung et al. 2015) but differs in the quantitative details (Supplementary Figs. S2A and S2B). This may be partly attributed to different myosin preparations used in the experiments and assumed in the simulations (one-headed vs two-headed; see legend of Supplementary Fig. S2 and “Discussion” section). As in (Capitanio et al. 2012), a new detachment process became important in simulations with imposed resistive loads. However, this process, associated with Pi-binding in the model, was appreciably slower than in the experiments and was not analyzed in detail (see further “Discussion” section).

**Fig. 3:**
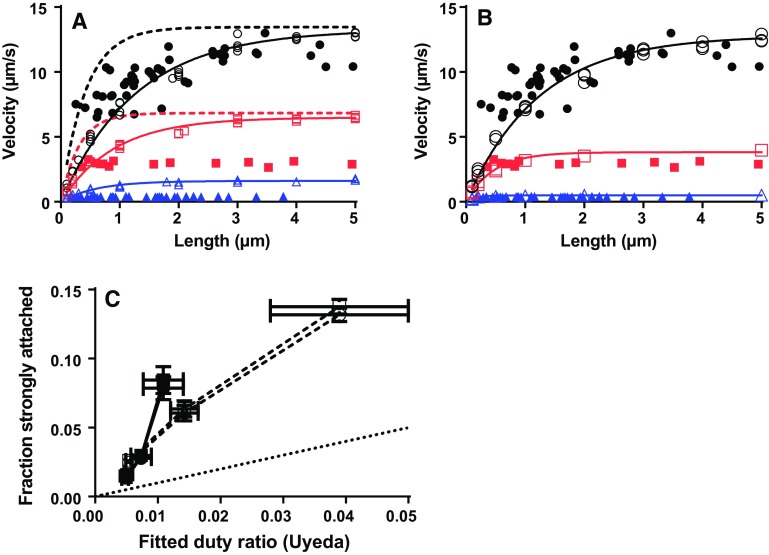
Velocity vs filament length in the in vitro motility assay. **a** Simulated velocity vs filament length (5000 active myosin heads per µm^2^) for standard conditions assuming K_w_ = 12 (*open symbols, full lines*) compared to experimental in vitro motility assay data (*filled symbols*; Persson et al. 2013). Simulated and experimental data at 1 mM (*black*), 0.1 mM (*red*) and 0.01 mM (*blue*) [MgATP]. *Filled lines* represent fits of Eq. 1 to the simulated data. *Dashed lines* represent fits of Eq. 1 to simulation results (not shown) where three independent myosin binding sites are assumed per 36 nm of the actin filament. **b** Simulation results and experimental results for similar conditions as in A (similar color and format coding of lines and symbols) but assuming non-linear cross-bridge elasticity (i.e. k_s_ = 2.8 pN/nm for x > 0 and k_s_ = 0.25 pN/nm otherwise). **c** Fraction of attached myosin heads in strongly bound states from simulations plotted against duty ratio from fit of Eq. 1 to the simulated data. The strongly bound heads in the simulations were either taken as all heads in the states AM*D_L_, AM*D_H_, AMD, AM and AMT (*squares*) or only the heads in the states AM*D_H_, AMD, AM and AMT (*circles*). The cross-bridge elasticity was either assumed to be linear (*filled symbols and full line*) or non-linear (*open symbols and dashed line*). Horizontal error bars are the 95% CIs obtained in the non-linear regression fit of Eq. 1 with maximum velocity and duty ratio as varying parameters in the regression analysis. *Dotted line* has slope 1. Similar data as in A and C for K_w_ = 1 are shown in Supplementary Fig. S3

The model reproduces key aspects of experimental in vitro motility assay results. The general shape of the relationship between velocity and the number of available myosin heads (N; proportional to filament length) is reasonably well predicted. If K_w_ = 1, the relationship is best predicted for different [MgATP] if the number (n_s_) of independent myosin-binding sites is close to 3 per 36 nm (Supplementary Fig. S3). If K_w_ is increased to 12, the experimental data are instead best predicted with only one myosin binding site per 36 nm (n_s_ = 1) (Fig. 3). In the following, K_w_ = 12 instead of K_w_ = 1 will be used as standard value (see further “Discussion” section) together with n_s_ = 1.

The predicted ATP turnover rate per head (19 s^−1^) in the in vitro motility assay is similar to experimental results (13 s^−1^ (Uyeda et al. 1990); see also Toyoshima et al. 1990) and constitutes a similar fraction of the actomyosin ATPase in solution (86 s^−1^ in experiments (Brenner and Eisenberg 1986); and 89 s^−1^ predicted here (see above).

In order to account for the high maximum velocity at high [MgATP] (Fig. 3) it was necessary to assume a strain-dependent MgATP-induced detachment rate (k_2_(x)). However, the appreciable strain-dependence required is not consistent with experimental findings (Capitanio et al. 2012; Nyitrai et al. 2006) and also leads to too high velocity at low [MgATP]. We therefore attempted to reproduce the data at both low and high [MgATP] with limited strain-dependence of k_2_(x) (cf. Fig. 4d in Capitanio et al. 2012). This was possible if it was also assumed that the cross-bridge elasticity is non-linear, with low stiffness of post-power-stroke cross-bridges (Kaya and Higuchi 2010). Such an idea was previously found to improve the fit to [MgATP]-velocity plots using a biochemically simpler, large-ensemble model (Persson et al. 2013). It can be seen (Fig. 3b; n_s_ = 1) that the effect of [MgATP] on maximum velocity is quite well accommodated without sacrificing faithful reproduction of the shape of the velocity–length relationships. This was achieved without other changes than reduced strain-dependence of k_2_(x) and introduction of non-linear cross-bridge elasticity.

Velocity-length data like those in Fig. 3 are often analyzed by fitting the equation of Uyeda et al. (Uyeda et al. 1990) (Eq. 1; lines in Fig. 3) to obtain an estimate of the duty ratio. The latter parameter is the fraction of the ATP-turnover time that a given myosin head spends strongly attached to actin or, in other words, the fraction of strongly attached myosin heads at a given time. Fits of Eq. 1 (Uyeda et al. 1990) to the simulated velocity-length data in Fig. 3 gave duty ratios that correlated positively with the number of attached myosin heads according to the simulations (Fig. 3c). However, the duty ratios from fits of Eq. 1 substantially underestimate (by two to sixfold) the fraction of attached heads both if linear and non-linear cross-bridge elasticity (see above) is assumed in the simulations. This is not surprising because elastic coupling effects are not taken into account in the Uyeda et al. (1990) equation. In contrast, elastic coupling is central in the present model as well as in previous models for large ensembles and some recent models for small ensembles (Hilbert et al. 2013; Pate and Cooke 1991; Walcott et al. 2012). That is, binding of myosin heads to actin, but also detachment and other transitions of attached myosin heads that change total motor force, influence the strain (and thereby strain-dependent kinetics) of other actin-attached myosin heads. This is implemented here by re-equilibrating forces following each state transition (see Supplementary Methods). The functional relationship between the duty ratio (from Eq. 1) and the number of attached heads at different [MgATP] was very similar for K_w_ = 1 and K_w_ = 12 (Supplementary Fig. S3).

**Fig. 4:**
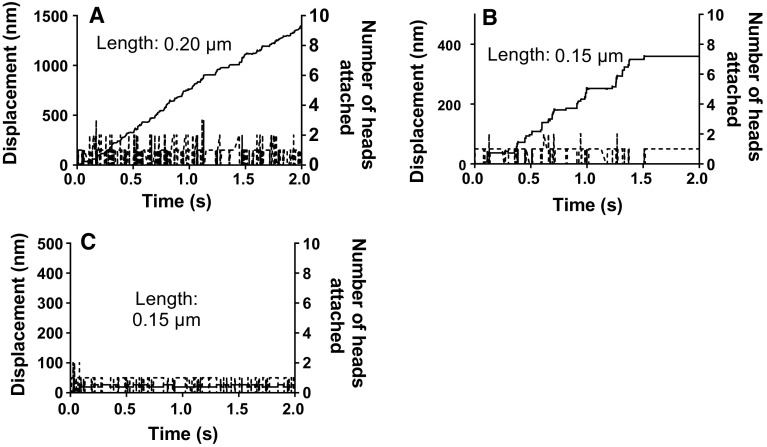
Plots of displacement (*left vertical axis; full line*) and number of attached myosin heads (*right axis; dashed line*) against time for filaments propelled by low number of myosin motors. **a** 0.20 µm long filament. Note, temporary stops in motility interspersed with phases of rapid displacement. **b** One example of simulation assuming 0.15 µm long filament. **c** Second example of 0.15 µm long filament. Note, switch in position upon myosin head attachment but no net displacement. Assumed motor density, 1000 µm^−2^ (see “Materials and Methods” section, text under Eq. 1) corresponding to 4–5 and 6 myosin heads available for interaction with 0.15 and 0.20 µm long filaments, respectively

Key results for short actin filaments and low myosin surface densities are also predicted (Fig. 4) such as the tendency for stops and pauses in motility, intervened by periods of rapid sliding when the number of available myosin heads is reduced below a certain critical value (Hilbert et al. 2013). Furthermore, in accordance with a minimum actin filament length of ~0.15 µm for maintained sliding at a myosin head density of 1000 µm^−2^ (Toyoshima et al. 1990), extended periods without attached myosin heads and/or without filament sliding (Fig. 4c), were observed in simulations under these assumptions.

**Fig. 5:**
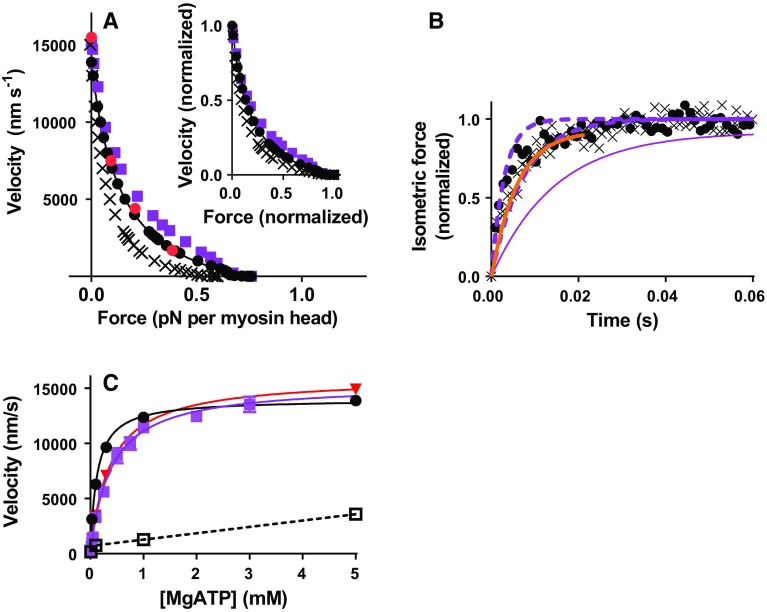
Predictions for large ensembles. **a** Simulated force–velocity data for muscle under standard conditions with K_w_ = 12 (*black circles*) or K_w_ = 1 (crosses). Approximate force–velocity relations deduced using different NEM-HMM/HMM ratios (*red*) also illustrated. The simulated standard data were fitted by the Hill (1938) hyperbolic equation (*black curve*). Data compared to experimental results (*purple* (Månsson et al. 1989); see text). Force normalized to the total number of available heads (whether attached to actin or not). For K_w_ = 12 and K_w_ = 1 average isometric force per attached myosin head was 6.95 pN and 6.42 pN, respectively. *Inset* Data replotted after normalization to maximum velocity and maximum force. **b** The rise of isometric force assuming maximum activation and standard conditions with K_w_ = 12 (filled circles; rate constant 245 ± 33 s^−1^ (mean ± 95% CI), in single exponential fit) and K_w_ = 1 (crosses; rate constant 152 ± 13 s^−1^). Simulations using Monte-Carlo approach with myosin head surface-density of 5000 µm^−2^ and filament length of 20 µm. Experimental fit (*full purple line*) of data from (Sleep et al. 2005) skinned fast rabbit skeletal muscle at 20 °C (ionic strength: 170 mM) adjusted to 30 °C, assuming Q_10_-value of 3 or 5 (*dashed lines*). Experimental data from intact fast skeletal muscle of the mouse at 30 °C (Månsson et al. 1989) also illustrated (*orange line*). **c** Velocity vs [MgATP]. Experimental data from in vitro motility assays (purple; fast rabbit skeletal muscle HMM, 30 °C, ionic strength 130 mM) from (Persson et al. 2013). It was argued (Persson et al. 2013) that these data are fully comparable to data from muscle cells. *Black filled line and symbols* simulations using standard parameter values and linear cross-bridge elasticity. *Red line and symbols* simulations using standard parameter values (K_w_ = 12) but assuming low cross-bridge stiffness (0.25 pN/nm) at negative strain (see text for details). Full curves represent Michaelis–Menten equation fitted to the data. Black: K_M_ = 0.123 ± 0.031 mM; V_max_ = 14 010 ± 830 nm/s. Purple: K_M_ = 0.387 ± 0.062 mM; V_max_ = 15 408 ± 715 nm/s. Red: K_M_ = 0.371± 0.27 mM; V_max_ = 16 010 ± 330 nm/s. Black open symbols and connecting dashed lines: Velocity vs [MgATP] assuming a total step length of 7.7 nm and on-times as in Fig. 2, i.e. using data from single molecule mechanics. *Note* Appreciably lower velocity at all [MgATP] than in simulations for large ensembles

Model predictions for large actomyosin ensembles were also compared to experimental data. First, force–velocity data (Fig. 5a) from fast mouse muscle at 30 °C [more complete, but otherwise similar to rabbit muscle data (Asmussen et al. 1994)] are well predicted and a small difference between the simulated and experimental curve is largely eliminated upon normalization (inset Fig. 5a). This is important because the force–velocity relationship reflects an intricate interplay between cross-bridge attachment and detachment kinetics as well as other features of cross-bridge operation (Albet-Torres et al. 2009; Duke 1999; Edman et al. 1997; Hill 1974; Huxley 1957; Piazzesi et al. 2007). The isometric force on average per attached cross-bridge was 6.95 pN if K_w_ = 12 and 6.42 pN if K_w_ = 1. These values are within the experimental errors of those (~ 6 pN) found in single molecule studies (Capitanio et al. 2012) using myosin preparations from fast mammalian muscle as well as in experiments on intact muscle fibers from the frog (Piazzesi et al. 2007). If K_w_ = 1, as suggested by K_M_ of the actomyosin ATPase (see above), the maximum power output (load x velocity) would be underestimated (crosses in Fig. 5a). The normalized force–velocity relationship would not be changed by increasing n_s_ from 1 to 3 (not shown) but the average force would increase threefold.

The simulated effects of altered [Pi] on isometric tension are approximately consistent with experimental data (Tesi et al. 2002) (Supplementary Fig. S1D). The prediction that changed [Pi] does not affect the maximum velocity (see Supplementary Fig. S1D for details) agrees with experimental findings under physiological pH (Caremani et al. 2013; Cooke and Pate 1985; Debold et al. 2011).

The time course of the isometric tension rise at full activation is well predicted (Fig. 5b) and is determined primarily by k_+P_(x), if the concentration of inorganic phosphate is low (Dantzig et al. 1992). If K_w_≈1, the rate of rise of force would be affected also by K_w_ because the quantity K_w_ k_+P_(x)/(K_w_ + 1) is the effective rate constant for transition from the MDP to the AM*D_L_ state. However, it can be seen in Fig. 5b that the effect on the rate of rise of isometric force of reducing K_w_ from 12 to 1 would be difficult to detect within the experimental uncertainty.

In accordance with experiments, the model predicts a nearly hyperbolic (Michaelis–Menten type) relationship between [MgATP] and velocity (Fig. 5c) but with lower K_M_^v^ value ([MgATP] at half maximum velocity) than in experiments. The latter deviation was eliminated, with maintained velocity at saturating [MgATP], by introduction of non-linear cross-bridge compliance (cf. Persson et al. 2013) together with reduced strain-dependence of k_2_(x) (Capitanio et al. 2012) (see also Fig. 3b). It is also of interest to compare the ensemble data in Fig. 5c to the [MgATP]–velocity relationship predicted by dividing the step length of 7.7 nm from single molecule data with the on-times from such data (Fig. 2). It is clear from the dashed line in Fig. 5c that the latter velocities are almost tenfold lower than those from ensemble simulations (cf. Brizendine et al. 2015).

**Fig. 6:**
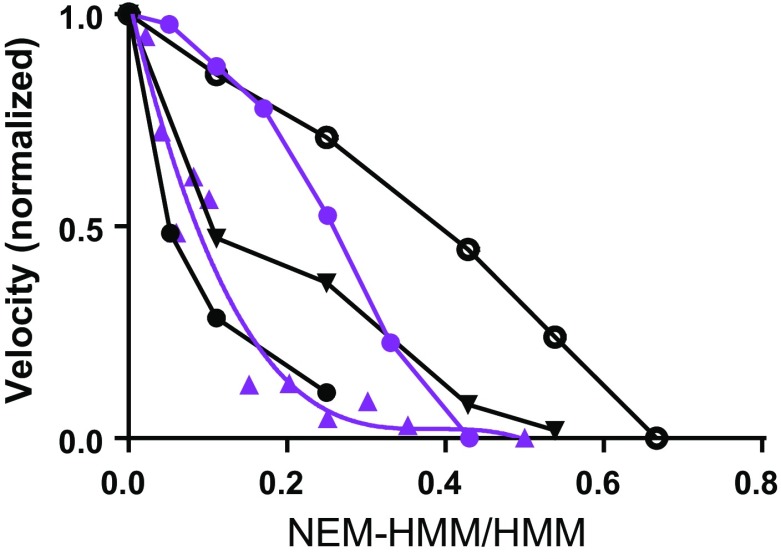
Sliding velocity in the in vitro motility assay vs [NEM-HMM]/[HMM] ratio where NEM-HMM is *N*-ethyl maleimide treated HMM. Simulated data (*black*) compared to experimental data (*purple*) from (Kim et al. 1996) (*circles*; fast rabbit HMM; 25 °C; ionic strength <50 mM) and (Amitani et al. 2001) (*triangles*, fitted with cubic polynomial; fast rabbit HMM, 25 °C; ionic strength <50 mM). Simulated behavior of long (20 µm) filaments assuming binding energy of NEM-HMM to actin of either 18 k_B_T (*filled circles*) or 7 k_B_T (*open circles*). The latter case also simulated for 1 µm long filaments (*triangles*). Note appreciable variability among experimental results but also variability among simulated data depending on exact conditions for the simulations. Experimental data points were obtained by measuring from figures in the cited papers

A frictional load is often imposed on myosin propelled filaments in the in vitro motility assay (“a loaded motility assay”) (Bing et al. 2000) by transient interaction of actin binding proteins on the surface with the actin filament. Simulations, assuming NEM-HMM to be the actin-binding protein, are consistent with experiments within the quite appreciable variability (Fig. 6). Furthermore, plots of velocities against the resistive loads produced by the NEM-HMM heads compare well to force–velocity data from simulations of forces developed during iso-velocity shortening (Fig. 5).

## Discussion

### General issues: relation to other studies

A key finding is that a classical model, of the Huxley–Hill type (Hill 1974; Huxley 1957) with detailed biochemical cycle (Fig. 1) approximates a range of contractile phenomena (e.g. Figs. 2, 3, 4, 5, 6; Supplementary Fig. S1) on experimental scales from single molecules to muscle, without considering details of muscle structure. Related, there is no need to assume emergent properties in the large ordered ensembles or to use different models for different conditions (e.g. Baker et al. 2002).

Steps towards including elastic coupling effects in the modelling of in vitro motility assay results have been taken previously (Baker et al. 2002; Duke 1999; Pate and Cooke 1991; Walcott et al. 2012; Vilfan and Duke 2003). Pate and Cooke (1991) used a slightly modified version of the (Huxley 1957) model to provide general insights into effects of varying number of motors but without detailed connection to key biochemical intermediates in actomyosin ATP turnover. The latter details are likely to be important in studies of pathologies but also for full understanding of normal function. More recent models have included additional details and provided further insight e.g. about the behavior of filaments at very low motor number (Hilbert et al. 2013).They have also reported effects of elastic coupling on the relationship between the number of motors and the average motor on-time, sliding distance and velocity (Baker et al. 2002; Walcott et al. 2012). However, unlike the present work, the previous studies did not consider all hierarchical levels from single molecules to muscle cells. Furthermore, the explicit separation of the Pi-release from both the main force-generating transition and the attachment step to actin, distinguishes the present model from most predecessors both for large (Månsson 2010; Pate and Cooke 1989; Persson et al. 2013) and small (Erdmann and Schwarz 2012; Hilbert et al. 2013; Pate and Cooke 1991; Walcott et al. 2012) ensembles. Whereas such separation is not essential to approximately fit the data in Fig. 5 (e.g. (Pate and Cooke 1989; Persson et al. 2013) the results demonstrate that a separate Pi-release and a realistic biochemical cycle does not compromise the capability of the model to account for a range of mechanical results on different scales (Figs. 2, 3, 4, 5, 6). This is important (e.g. Malnasi-Csizmadia and Kovacs 2010; Månsson et al. 2015) in order for a model to be useful for connecting biochemical transitions to mechanical events in fundamental studies and for elucidating effects of disease-causing mutations and effects of drugs. A separate Pi-release seems essential to account for structural and biochemical findings (Llinas et al. 2015; Sweeney and Houdusse 2010). Further, it seems required to account for findings in muscle fiber studies, e.g. the tension response of changes in temperature (temperature transients) (Ranatunga 2010) and to rapid changes in the concentration of inorganic phosphate (phosphate transients) (Dantzig et al. 1992).

Directly related to the above issue, the present study also differs from most earlier model studies by explicitly considering a weakly bound actomyosin state (AMDP) that is distinguished from a start of the power-stroke state without Pi (AM*D_L_). The properties of the weakly bound AMDP state have potentially important implications motivating that it is considered separately. For instance, different affinities of this state to actin noticeably affect (Fig. 5A) the force–velocity relationship. Furthermore, cross-bridges in the weakly bound state may also impose a viscoelastic load on actin filaments with a magnitude that varies with the experimental conditions and possibly between muscle cells and in vitro motility assays (Homsher et al. 1992) (however see Vikhorev et al. 2008). Finally, certain myosin head mutations [e.g. positively charged amino acids in surface loops (Colegrave and Peckham 2014)] may affect the weak-binding properties. It is therefore important to include the weakly bound state in a model intended to be useful for studies of diseases and drug development. Uncertainties regarding the actin affinity in the weak-binding state and their implications are considered below.

The present model overcomes existing barriers in data interpretation by allowing integration of results from experimental systems on different scales. Particularly, mechanical and kinetic results obtained using isolated proteins, e.g. solution studies, studies of single molecules or in vitro motility assays (Aksel et al. 2015; Sung et al. 2015; Wang et al. 2013), may be readily extrapolated to contractile properties of muscle. This will aid mechanistic insights into the physiology, pathology and pharmacology of striated muscle contraction. Whereas the model is largely based on states derived from mechanical experiments and biochemical kinetics, the states have coarse-grain structural equivalents, e.g. with lever arm up or down (insets of Fig. 1b) and the actin-binding cleft open or closed. Therefore, the model should be useful for correlating atomic resolution structural data (e.g. reviewed in Sweeney and Houdusse (2010), Månsson et al. (2015)) and contractile function from single molecules to large actomyosin ensembles.

For studies of pathologies, additional states, e.g. the so called super-relaxed state (Hooijman et al. 2011; Linari et al. 2015) may be important to include as well as effects of regulatory proteins and time varying activation. Inclusion of new states into the model is trivial. It is more complex to include time varying activation. This property is attributed to regulatory/modulatory proteins associated both with the thin filaments (troponin, tropomyosin) and the thick filaments, e.g. myosin regulatory light chains and myosin binding protein C (reviews in Kamm and Stull 2011; Moore et al. 2016; van Dijk et al. 2014). The issue may be treated either phenomenologically by time varying values of rate constants (Homsher et al. 2000; Månsson 2014; Webb et al. 2013) or explicitly using expanded Monte-Carlo approaches (Mijailovich et al. 2012; Rice and de Tombe 2004). However, due to computation time constraints, the latter approach will require extensive optimization for speed both of computational platforms and algorithms.

The effect of lengthening during activity is not in focus here. However, this is an important physiological phenomenon that also needs to be taken into account when studying e.g. pathologies (Månsson 2014). A simple approach to account for key aspects of the mechanics of lengthening contraction is therefore illustrated in Supplementary Fig. S4. However, undoubtedly, both effects of other proteins than actin and myosin e.g. titin, and emergent properties related to sarcomere non-uniformities will need to be considered in a complete treatment (Campbell et al. 2011; Edman 2012; Higuchi and Umazume 1985; Rassier 2012a; Wang et al. 1984).

### Limitations of the model: related biological insights and suggestions for further studies

As mentioned above, no attempts were made to fit conditions of time-varying activation or lengthening contractions. However, additional experimental results are not accounted for in full quantitative detail. This may either be attributed to experimental uncertainties or limitations in the model representation of biological mechanisms. Such limitations necessarily exist in any model and are important because they point out poorly understood mechanisms and prompt testing of alternative ideas. Below, the discrepancy between predictions of the present model and experimental data is considered in relation to experimental uncertainties and alternative models.

One limitation related to poorly understood characteristics of the cross-bridge elasticity (whether it is linear or non-linear) is the requirement for significantly greater strain/load dependence of k_2_ in the model than suggested by the load-dependence of the ATP-induced detachment in single molecule measurements (Capitanio et al. 2012; Supplementary Fig. S2). This was necessary to account for the high maximal velocity at saturating [MgATP] but led to overestimation of velocity at lower [MgATP] (with too low K_M_^v^-value). Interestingly, however, both the magnitudes of the maximum velocity and K_M_^v^ for the [MgATP]–v relationship are accounted for with very low (or no) strain dependence of k_2_ if the cross-bridge compliance for x < 0 nm is assumed to be ~1/10 of that at x > 0 nm, simulating non-linear cross-bridge elasticity (Kaya and Higuchi 2010). On the other hand, evidence suggesting a non-linear elasticity has been obtained only in certain in vitro assays (Brizendine et al. 2015; Kaya and Higuchi 2010; Persson et al. 2013) and there is currently no firm evidence that the cross-bridge elasticity is non-linear in muscle cells. Clearly, the latter issue will be of outmost importance to clarify.

One further problem is that the detailed course of events associated with transitions between the MDP and the AM*D_L_ states cannot be represented in full detail due to ambiguities in available experimental data (reviewed in Månsson et al. 2015). These poorly understood details might account for the failure to perfectly reproduce the relationship between [Pi] and isometric tension as well as for lack of key ATP-independent detachment processes in single molecule data (Capitanio et al. 2012). The latter findings seem to suggest additional states between the AMDP and the AM*D_L_ states. Here, it is of interest to consider ideas (Caremani et al. 2013; Ford et al. 1986; Geeves et al. 1984; Smith and Mijailovich 2008) that mechanical transitions between high force and low force conformations occur in different biochemical states. For instance, a mechanical transition (similar to that from the AM*D_L_ to AM*D_H_ state) may occur also in an AM*DP biochemical state. This would add a high-force state from which rapid ATP independent detachment may occur. Additionally, it would fit with at least two tensing steps as suggested recently (Offer and Ranatunga 2013) and it may fit into a framework to explain high maximum power-output of muscle (Caremani et al. 2013) without the need for a high value of K_w_ as used here. However, again, the available experimental data is ambiguous with regard to both the existence and the properties of these postulated states and transitions (Månsson et al. 2015).

One additional reason for discrepancies between the model predictions and experimental results (Capitanio et al. 2012) for load-dependence of cross-bridge detachment (Supplementary Fig. S2) could be that the model is defined primarily on the basis of data for two-headed myosin whereas the experiments use one-headed myosin. Peculiarities in relation to other experimental results (see Supplementary Fig. S2) lend support to that idea.

As discussed above, the weakly bound myosin state is not included as a separate state in most models. The striking effects of a tenfold change in actin affinity is therefore of interest. For instance, the need to assume a higher value of K_w_ to account for high power-output of muscle than expected from actin-binding in solution (association constant K_a_), could mean that cooperative or other phenomena operate during shortening against intermediate loads (Caremani et al. 2013; Edman et al. 1997; Månsson 2010). On the other hand, there may be some uncertainty (Brenner et al. 1986; Eisenberg et al. 1980) in the effective actin concentration, in the muscle sarcomere, which relates K_w_ to K_a_. Alternatively, the affinity of weakly bound actomyosin cross-bridges might vary between conditions as suggested for actomyosin bonds in other states (Guo and Guilford 2006). Further, as an additional alternative, the surface attachment of myosin motors, either to the motility assay surface or the thick filament backbone, may account for increased affinity of the weakly bound actomyosin state in mechanical experiments. It would therefore be valuable if current estimates of the affinity of weakly bound heads with myosin tethered on a surface are verified under physiological conditions and full activation.

### Implications for the interpretation of in vitro motility assay data at different motor densities

In terms of the model of Uyeda et al.(1990) (Eq. 1) the velocity varies with the number (N) of available motors due to the fraction of time that at least one motor propels the actin filament; no elastic coupling effects between motors are considered as in classical muscle models (Hill 1974; Huxley 1957). The present study and the results in recent work (Walcott et al. 2012) strongly suggest that, if such elastic coupling is present, the parameter values obtained in fits of Eq. 1 (Uyeda et al. 1990) do not adequately describe the system. This is important because of the extensive use of the Uyeda model (Uyeda et al. 1990) for analysis of in vitro motility assay data. The present results (Fig. 3) accord with 1–3 myosin binding sites per 36 nm of the actin filament which indicates that myosin heads only bind to target zones at 36 nm spacing. This accords with single molecule mechanics data (Capitanio et al. 2006; Steffen et al. 2001) but is generally not considered in the analysis of in vitro motility assays for small ensembles.

### Relation to engineering approaches involving actomyosin

An interesting development in the recent decade is engineering of myosin motors to incorporate radically new functionalities, e.g. with regard to the movement direction along actin filaments (Tsiavaliaris et al. 2004) or even the remote switching of this directionality, e.g. by illumination (Nakamura et al. 2014). In addition to potential uses in nanotechnological applications (Kumar et al. 2016; Månsson 2012; Nicolau et al. 2016) the engineering results pose new questions about how certain functions relate to structural elements in the motor. The present model, by virtue of coarse-grain structural equivalence of different states, would be useful for elucidating how the engineered elements give rise to some of their functional consequences. Conversely, the model may also be useful for guiding engineering of motors with certain properties, e.g. for use in nanotechnology.

## Conclusions

The results demonstrate that actomyosin contractile function, from single molecules to large ensembles, is reasonably well approximated by one unifying chemo-mechanical model with a realistic biochemical cycle. Observed limitations in model predictions identify needs for new experimental studies. This includes the transitions between the MDP state over the weak-binding actomyosin state to the start of power-stroke AM*D_L_ state, i.e. the properties of the weakly bound actomyosin state and the temporal relationship between Pi-release and the main force-generating transition. Furthermore, detailed characterization of the cross-bridge elasticity is essential. Particularly, it is important to clarify if non-linear elasticity is limited to certain in vitro assays or is present in living muscle. A future version of the model for analysis of diseases and drug effects would benefit from new experimental evidence to fully clarify the mentioned issues. Finally, the study has also given new insights important for the interpretation of in vitro motility assays and for engineering of motors.

## Electronic supplementary material

Below is the link to the electronic supplementary material.

Supplementary material 1 (DOCX 4167 kb)

## References

[CR1] Aksel T, Choe YuE, Sutton S, Ruppel KM, Spudich JA (2015). Ensemble force changes that result from human cardiac myosin mutations and a small-molecule effector. Cell Rep.

[CR2] Albet-Torres N (2009). Drug effect unveils inter-head cooperativity and strain-dependent ADP release in fast skeletal actomyosin. J Biol Chem.

[CR3] Amitani I, Sakamoto T, Ando T (2001). Link between the enzymatic kinetics and mechanical behavior in an actomyosin motor. Biophys J.

[CR4] Amrute-Nayak M (2010). Targeted Optimization of a protein nanomachine for operation in biohybrid devices. Angew Chem Int Ed.

[CR5] Asmussen G, Beckers-Bleukx G, Marechal G (1994). The force–velocity relation of the rabbit inferior oblique muscle; influence of temperature. Pflugers Arch Eur J Physiol.

[CR6] Baker JE, Brosseau C, Joel PB, Warshaw DM (2002). The biochemical kinetics underlying actin movement generated by one and many skeletal muscle Myosin molecules. Biophys J.

[CR7] Batters C, Veigel C, Homsher E, Sellers JR (2014). To understand muscle you must take it apart. Front Physiol.

[CR8] Bing W, Knott A, Marston SB (2000). A simple method for measuring the relative force exerted by myosin on actin filaments in the in vitro motility assay: evidence that tropomyosin and troponin increase force in single thin filaments. Biochem J.

[CR9] Brenner B, Eisenberg E (1986). Rate of force generation in muscle: correlation with actomyosin ATPase activity in solution. Proc Natl Acad Sci USA.

[CR10] Brenner B, Yu LC, Greene LE, Eisenberg E, Schoenberg M (1986). Ca^2+^-sensitive cross-bridge dissociation in the presence of magnesium pyrophosphate in skinned rabbit psoas fibers. Biophys J.

[CR11] Brizendine RK, Alcala DB, Carter MS, Haldeman BD, Facemyer KC, Baker JE, Cremo CR (2015). Velocities of unloaded muscle filaments are not limited by drag forces imposed by myosin cross-bridges. Proc Natl Acad Sci USA.

[CR12] Brunello E (2007). Skeletal muscle resists stretch by rapid binding of the second motor domain of myosin to actin. Proc Natl Acad Sci USA.

[CR13] Campbell KS (2009). Interactions between connected half-sarcomeres produce emergent mechanical behavior in a mathematical model of muscle. PLoS Comp Biol.

[CR14] Campbell SG, Hatfield PC, Campbell KS (2011). A mathematical model of muscle containing heterogeneous half-sarcomeres exhibits residual force enhancement. PLoS Comp Biol.

[CR15] Capitanio M (2006). Two independent mechanical events in the interaction cycle of skeletal muscle myosin with actin. Proc Natl Acad Sci USA.

[CR16] Capitanio M (2012). Ultrafast force-clamp spectroscopy of single molecules reveals load dependence of myosin working stroke. Nature Methods.

[CR17] Caremani M, Melli L, Dolfi M, Lombardi V, Linari M (2013). The working stroke of the myosin II motor in muscle is not tightly coupled to release of orthophosphate from its active site. J Physiol.

[CR18] Colegrave M, Peckham M (2014). Structural implications of beta-cardiac myosin heavy chain mutations in human disease. Anat Rec (Hoboken).

[CR19] Cooke R, Pate E (1985). The effects of ADP and phosphate on the contraction of muscle fibers. Biophys J.

[CR20] Dantzig JA, Goldman YE, Millar NC, Lacktis J, Homsher E (1992). Reversal of the cross-bridge force-generating transition by photogeneration of phosphate in rabbit psoas muscle fibres. J Physiol.

[CR21] Debold EP, Turner MA, Stout JC, Walcott S (2011). Phosphate enhances myosin-powered actin filament velocity under acidic conditions in a motility assay. Am J Physiol Regul Integr Comp Physiol.

[CR22] Duke TA (1999). Molecular model of muscle contraction. Proc Natl Acad Sci USA.

[CR23] Edman KAP (2012). Residual force enhancement after stretch in striated muscle. A consequence of increased myofilament overlap?. J Physiol.

[CR24] Edman KAP, Flitney FW (1982). Laser diffraction studies of sarcomere dynamics during `isometric’ relaxation in isolated muscle fibres of the frog. J Physiol (Lond).

[CR25] Edman KAP, Månsson A, Caputo C (1997). The biphasic force–velocity relationship in frog muscle fibres and its evaluation in terms of cross-bridge function. J Physiol (Lond).

[CR26] Eisenberg E, Hill TL (1978). A cross-bridge model of muscle contraction. Prog Biophys Mol Biol.

[CR27] Eisenberg E, Hill TL, Chen Y (1980). Cross-bridge model of muscle contraction. Quant Anal Biophys J.

[CR28] Erdmann T, Schwarz US (2012). Stochastic force generation by small ensembles of myosin II motors. Phys Rev Lett.

[CR29] Ferrantini C, Belus A, Piroddi N, Scellini B, Tesi C, Poggesi C (2009). Mechanical and energetic consequences of HCM-causing mutations. J Cardiovasc Transl Res.

[CR30] Finer JT, Simmons RM, Spudich JA (1994). Single myosin molecule mechanics: piconewton forces and nanometre steps. Nature.

[CR31] Ford LE, Huxley AF, Simmons RM (1986). Tension transients during the rise of tetanic tension in frog muscle fibres. J Physiol (Lond).

[CR32] Frey N, Luedde M, Katus HA (2012). Mechanisms of disease: hypertrophic cardiomyopathy. Nat Rev Cardiol.

[CR33] Geeves MA, Goody RS, Gutfreund H (1984). Kinetics of acto-S1 interaction as a guide to a model for the crossbridge cycle. J Muscle Res Cell Motil.

[CR34] Gillespie DT (1976). A general method for numerically simulating the stochastic time evolution of coupled chemical reactions. J Comput Phys.

[CR35] Gordon AM, Homsher E, Regnier M (2000). Regulation of contraction in striated muscle. Physiol Rev.

[CR36] Green EM (2016). A small-molecule inhibitor of sarcomere contractility suppresses hypertrophic cardiomyopathy in mice. Science.

[CR37] Guo B, Guilford WH (2006). Mechanics of actomyosin bonds in different nucleotide states are tuned to muscle contraction. Proc Natl Acad Sci USA.

[CR38] Higuchi H, Umazume Y (1985). Localization of the parallel elastic components in frog skinned muscle fibers studied by the dissociation of the A- and I-bands. Biophys J.

[CR39] Hilbert L, Cumarasamy S, Zitouni NB, Mackey MC, Lauzon AM (2013). The kinetics of mechanically coupled myosins exhibit group size-dependent regimes. Biophys J.

[CR190] Hill TL (1938). The heat of shortening and the dynamic constants of muscle. Proc Royal Soc B.

[CR40] Hill TL (1974). Theoretical formalism for the sliding filament model of contraction of striated muscle. Part I. Prog Biophys Mol Biol.

[CR41] Homsher E, Wang F, Sellers JR (1992). Factors affecting movement of F-actin filaments propelled by skeletal muscle heavy meromyosin. Am J Physiol.

[CR42] Homsher E, Lee DM, Morris C, Pavlov D, Tobacman LS (2000). Regulation of force and unloaded sliding speed in single thin filaments: effects of regulatory proteins and calcium. J Physiol.

[CR43] Hooijman P, Stewart MA, Cooke R (2011). A new state of cardiac myosin with very slow ATP turnover: a potential cardioprotective mechanism in the heart. Biophys J.

[CR44] Huxley AF (1957). Muscle structure and theories of contraction. Prog Biophys Biophys Chem.

[CR45] Huxley AF, Simmons RM (1971). Proposed mechanism of force generation in striated muscle. Nature.

[CR46] Ishigure Y, Nitta T (2015). Simulating an actomyosin in vitro motility assay: toward the rational design of actomyosin-based microtransporters. IEEE Trans Nanobiosci.

[CR47] Ishijima A, Kojima H, Higuchi H, Harada Y, Funatsu T, Yanagida T (1996). Multiple- and single-molecule analysis of the actomyosin motor by nanometer-piconewton manipulation with a microneedle: unitary steps and forces. Biophys J.

[CR48] Kalganov A, Shalabi N, Zitouni N, Kachmar LH, Lauzon AM, Rassier DE (2013). Forces measured with micro-fabricated cantilevers during actomyosin interactions produced by filaments containing different myosin isoforms and loop 1 structure. Biochim Biophys Acta.

[CR49] Kamm KE, Stull JT (2011). Signaling to myosin regulatory light chain in sarcomeres. J Biol Chem.

[CR50] Kaya M, Higuchi H (2010). Nonlinear elasticity and an 8-nm working stroke of single myosin molecules in myofilaments. Science.

[CR51] Kim E, Miller CJ, Reisler E (1996). Polymerization and in vitro motility properties of yeast actin: a comparison with rabbit skeletal alpha-actin. Biochemistry.

[CR52] Kohler J (2003). Familial hypertrophic cardiomyopathy mutations in troponin I (K183D, G203S, K206Q) enhance filament sliding. Physiol Genomics.

[CR53] Kron SJ, Spudich JA (1986). Fluorescent actin-filaments move on myosin fixed to a glass-surface. Proc Natl Acad Sci USA.

[CR54] Kron SJ, Toyoshima YY, Uyeda TQ, Spudich JA (1991). Assays for actin sliding movement over myosin-coated surfaces. Methods Enzymol.

[CR55] Kubalek EW, Uyeda TQ, Spudich JA (1992). A Dictyostelium myosin II lacking a proximal 58-kDa portion of the tail is functional in vitro and in vivo. Mol Biol Cell.

[CR56] Kumar S (2016). Sensing protein antigen and microvesicle analytes using high-capacity biopolymer nano-carriers. Analyst.

[CR57] Li M, Larsson L (2010). Force-generating capacity of human myosin isoforms extracted from single muscle fibre segments. J Physiol.

[CR58] Linari M (2015). Force generation by skeletal muscle is controlled by mechanosensing in myosin filaments. Nature.

[CR59] Llinas P (2015). How actin initiates the motor activity of Myosin. Dev Cell.

[CR60] Malik FI (2011). Cardiac myosin activation: a potential therapeutic approach for systolic heart failure. Science.

[CR61] Malnasi-Csizmadia A, Kovacs M (2010). Emerging complex pathways of the actomyosin powerstroke. Trends Biochem Sci.

[CR62] Månsson  A (2010). Actomyosin-ADP states, inter-head cooperativity and the force–velocity relation of skeletal muscle. Biophys J.

[CR63] Månsson  A (2014). Hypothesis and theory: mechanical instabilities and non-uniformities in hereditary sarcomere myopathies. Front Physiol.

[CR64] Månsson A (2012). Translational actomyosin research: fundamental insights and applications hand in hand. J Muscle Res Cell Motil.

[CR65] Månsson  A, Morner J, Edman KA (1989). Effects of amrinone on twitch, tetanus and shortening kinetics in mammalian skeletal muscle. Acta Physiol Scand.

[CR66] Månsson A, Rassier D, Tsiavaliaris G (2015). Poorly understood aspects of striated muscle contraction. BioMed Research Int.

[CR67] Marston SB (2011). How do mutations in contractile proteins cause the primary familial cardiomyopathies?. J Cardiovasc Transl Res.

[CR68] McKillop DF, Geeves MA (1993). Regulation of the interaction between actin and myosin subfragment. 1: Evidence for three states of the thin filament. Biophys J.

[CR69] Mijailovich SM, Kayser-Herold O, Li X, Griffiths H, Geeves MA (2012). Cooperative regulation of myosin-S1 binding to actin filaments by a continuous flexible Tm–Tn chain. Eur Biophys J.

[CR70] Moore JR, Campbell SG, Lehman W (2016). Structural determinants of muscle thin filament cooperativity. Arch Biochem Biophys.

[CR71] Nakamura M, Chen L, Howes SC, Schindler TD, Nogales E, Bryant Z (2014). Remote control of myosin and kinesin motors using light-activated gearshifting. Nat Nanotechnol.

[CR72] Nicolau DV (2016). Parallel computation with molecular-motor-propelled agents in nanofabricated networks. Proc Natl Acad Sci USA.

[CR73] Nishizaka T, Seo R, Tadakuma H, Kinosita K, Ishiwata S (2000). Characterization of single actomyosin rigor bonds: load dependence of lifetime and mechanical properties. Biophys J.

[CR74] Nyitrai M, Rossi R, Adamek N, Pellegrino MA, Bottinelli R, Geeves MA (2006). What limits the velocity of fast-skeletal muscle contraction in mammals?. J Mol Biol.

[CR75] Offer G, Ranatunga KW (2013). A cross-bridge cycle with two tension-generating steps simulates skeletal muscle mechanics. Biophys J.

[CR77] Pate E, Cooke R (1989). A model of crossbridge action: the effects of ATP, ADP and Pi. J Muscle Res Cell Motil.

[CR78] Pate E, Cooke R (1991). Simulation of stochastic processes in motile crossbridge systems. J Muscle Res Cell Motil.

[CR79] Persson M, Bengtsson E, ten Siethoff L, Månsson  A (2013). Nonlinear cross-bridge elasticity and post-power-stroke events in fast skeletal muscle actomyosin. Biophys J.

[CR80] Piazzesi G (2007). Skeletal muscle performance determined by modulation of number of Myosin motors rather than motor force or stroke size. Cell.

[CR81] Ranatunga KW (2010). Force and power generating mechanism(s) in active muscle as revealed from temperature perturbation studies. J Physiol.

[CR82] Rassier DE (2012). The mechanisms of the residual force enhancement after stretch of skeletal muscle: non-uniformity in half-sarcomeres and stiffness of titin. Proc Royal Soc B.

[CR83] Rassier DE (2012). Residual force enhancement in skeletal muscles: one sarcomere after the other. J Muscle Res Cell Motil.

[CR84] Rice JJ, de Tombe PP (2004). Approaches to modeling crossbridges and calcium-dependent activation in cardiac muscle. Prog Biophys Mol Biol.

[CR85] Schindler TD, Chen L, Lebel P, Nakamura M, Bryant Z (2014). Engineering myosins for long-range transport on actin filaments. Nat Nanotechnol.

[CR86] Sleep J, Irving M, Burton K (2005). The ATP hydrolysis and phosphate release steps control the time course of force development in rabbit skeletal muscle. J Physiol.

[CR87] Smith DA, Mijailovich SM (2008). Toward a unified theory of muscle contraction. II: predictions with the mean-field approximation. Ann Biomed Eng.

[CR88] Sommese RF (2013). Molecular consequences of the R453C hypertrophic cardiomyopathy mutation on human beta-cardiac myosin motor function. Proc Natl Acad Sci USA.

[CR89] Song W (2011). Molecular mechanism of the E99K mutation in cardiac actin (ACTC Gene) that causes apical hypertrophy in man and mouse. J Biol Chem.

[CR90] Spudich JA (2014). Hypertrophic and dilated cardiomyopathy: four decades of basic research on muscle lead to potential therapeutic approaches to these devastating genetic diseases. Biophys J.

[CR91] Steffen W, Smith D, Simmons R, Sleep J (2001). Mapping the actin filament with myosin. Proc Natl Acad Sci USA.

[CR92] Steinmetz PR (2012). Independent evolution of striated muscles in cnidarians and bilaterians. Nature.

[CR93] Sung J (2015). Harmonic force spectroscopy measures load-dependent kinetics of individual human beta-cardiac myosin molecules. Nature Commun.

[CR94] Sweeney HL, Houdusse A (2010). Structural and functional insights into the Myosin motor mechanism. Annu Rev Biophys.

[CR95] Tanner BC, Daniel TL, Regnier M (2007). Sarcomere lattice geometry influences cooperative myosin binding in muscle. PLoS Comp Biol.

[CR96] Teekakirikul P, Padera RF, Seidman JG, Seidman CE (2012). Hypertrophic cardiomyopathy: translating cellular cross talk into therapeutics. J Cell Biol.

[CR97] Teerlink JR (2011). Dose-dependent augmentation of cardiac systolic function with the selective cardiac myosin activator, omecamtiv mecarbil: a first-in-man study. Lancet.

[CR98] Tesi C, Colomo F, Piroddi N, Poggesi C (2002). Characterization of the cross-bridge force-generating step using inorganic phosphate and BDM in myofibrils from rabbit skeletal muscles. J Physiol.

[CR99] Toyoshima YY, Kron SJ, Spudich JA (1990). The myosin step size: measurement of the unit displacement per ATP hydrolyzed in an in vitro assay. Proc Natl Acad Sci USA.

[CR100] Tsiavaliaris G, Fujita-Becker S, Manstein DJ (2004). Molecular engineering of a backwards-moving myosin motor. Nature.

[CR101] Uyeda TQ, Kron SJ, Spudich JA (1990). Myosin step size. Estimation from slow sliding movement of actin over low densities of heavy meromyosin. J Mol Biol.

[CR102] van Dijk SJ, Bezold KL, Harris SP (2014). Earning stripes: myosin binding protein-C interactions with actin. Pflugers Arch.

[CR103] Vikhorev PG, Vikhoreva NN, Månsson  A (2008). Bending flexibility of actin filaments during motor-induced sliding. Biophys J.

[CR104] Vilfan A, Duke T (2003). Instabilities in the transient response of muscle. Biophys J.

[CR105] Walcott S, Warshaw DM, Debold EP (2012). Mechanical coupling between myosin molecules causes differences between ensemble and single-molecule measurements. Biophys J.

[CR106] Wang K, Ramirez-Mitchell R, Palter D (1984). Titin is an extraordinarily long, flexible, and slender myofibrillar protein. Proc Natl Acad Sci USA.

[CR107] Wang Y, Ajtai K, Burghardt TP (2013). The Qdot-labeled actin super-resolution motility assay measures low-duty cycle muscle myosin step size. Biochemistry.

[CR108] Webb M, del Jackson R, Stewart TJ, Dugan SP, Carter MS, Cremo CR, Baker JE (2013). The myosin duty ratio tunes the calcium sensitivity and cooperative activation of the thin filament. Biochemistry.

[CR109] Woledge RC, Curtin NA, Homsher E (1985) Energetic aspects of muscle contraction. Monographs of the physiological society No. 41. Academic Press, London 3843415

