# Supplementary material for: Actomyosin based contraction: one mechanokinetic model from single molecules to muscle?
Source: J Muscle Res Cell Motil. 2016 Nov 18;37(6):181–94. doi: 10.1007/s10974-016-9458-0 (PMC5383694; doi:10.1007/s10974-016-9458-0)
Supplement: Supplementary file 1 — Supplementary material 1 (DOCX 4167 kb) [file 10974_2016_9458_MOESM1_ESM.docx]

**Supplementary Material**

**Actomyosin based contraction – one mechanokinetic model from single molecules to muscle?**

By

Alf Månsson

**Supplementary Theory**

*ATP-hydrolysis, cross-bridge attachment, phosphate release and major force-generating transition*

The recovery stroke and the hydrolysis of ATP to ADP and inorganic phosphate (Pi) on the active site of myosin is lumped together into one transition separating the MT and MDP states. This transition is governed by a rate constant k_3_ which, as well as its reversal k-_3_, is independent of x.

The equilibrium between detached myosin heads in the MDP state and myosin heads in non-stereospecifically weak-binding AMDP state is governed by an equilibrium constant K_w_(x) given by:

K_w_(x)=exp($\Delta$G_w_-(ksw/2)(x-x_w_)^2^/k_B_T) if x∈[-1.3,16.7]nm (S1a)

K_w_(x)=0 for other x-values (S1b)

Here, ksw is the stiffness of myosin cross-bridges in the AMDP state and x_w_ is the x-value for the minimum in the free energy of binding ($\Delta$G_w_ ; in units of k_B_T) of the weakly bound state (Table S1). The elastic energy in the exponent is divided by k_B_T (4 pN nm) in order to transform it into units of k_B_T. The invididual rates are either treated as infinitely high (Monte-Carlo simulations) or are assumed to be very high in simulation for large ensembles based on solution of differential equations. In the latter case the rates were taken to be of the order of 10 000 s^-1^ at very low velocities (isometric) and 1000 000 s^-1^ at near maximum shortening velocity.

The transition from the weakly and non-stereospecifically bound AMDP state to the phosphate free and stereospecifically bound AM*D_L_ state, is governed by the rate function:

k_a_(x)≈k_+P_(x)=k_b0_exp[$\Delta$G_AMDP-AM*DP_–(ks/2)(x-x_1_)^2^/(2k_B_T)+(ksw/2)(x-x_w_)^2^/(2 k_B_T)] (S2)

The values of the parameters k_b_,$\Delta$G_AMDP-AM*DP,_ ks and x_1_ are given in Tables S1-S2. The reversal of this transition (Eq. S2), including the P_i_ binding to the AMD_L_ state is governed by the rate function:

k_-P_(x)=k_b_(x) [Pi]/(K_C_+[Pi]) (S3)

where [P_i_] is the concentration of inorganic phosphate, K_C_ is the phosphate dissociation constant and:

k_b_(x)= k_b0_ exp[(ks/2)(x-x_1_)^2^/(2k_B_T) - ksw(x-x_w_)^2^/(2k_B_T)] (S4)

Here, the AM*DP-state is an intermediate, potentially force-producing state ([Dantzig et al. 1992](#_ENREF_7)) (between the AMDP and the AM*D_L_ states).

The main force-generating transition i.e. the power-stroke or tensing step is assumed to be a rapid equilibrium governed by the equilibrium constant:

K_LH_(x)=k_LH+_(x)/k_LH-_(x) (S5)

where

k_LH+_(x)=k_LH-_(x) exp($\Delta$G_AM*DL-AM*DH_ +ks(x-x_1_)^2^/(2k_B_T)-ks(x-x_2_)^2^/(2k_B_T)) (S6)

and

k_LH-_(x)=2000 s^-1^ (S7)

If K_LH_ (i)>1000 according to Eq. S5, it was set equal to 1000 in Monte-Carlo simulations.

The individual rate constants were taken as infinitely high in the Monte-Carlo simulations, i.e. only the equilibrium constant for the reaction is considered. Due to the high rates involved, certain simulations would, otherwise, become computationally untractable e.g. simulation of in vitro motility assays for long myosin filaments (e.g. ≥ 5 µm) and saturating myosin motor densities. To allow comparison between Monte-Carlo simulations and simulations based on solution of Master equations, high maximum values (at large negative x) were allowed for the individual forward rates (k_LH+_(x)) in the latter simulations. These values were limited to 10 000 s^-1^ under near isometric conditions and 10 000 000 s^-1^ near maximum shortening velocity. Higher values would cause instabilities in numerical solution of the differential equations.

Clearly, the above treatment of the main force-generating transition as an instantantaneous equilibrium makes it impossible to simulate the detailed time courses of rapid transients (e.g. tension transients ([Huxley and Simmons 1971](#_ENREF_12))). Importantly, transient responses would be possible to simulate using the present model if realistic rates are included for the individual transitions in the rapid equilibria. However, this would considerably slow the Monte-Carlo simulations and would therefore be possible only for very brief simulation runs.

*Cross-bridge detachment at the end of the power-stroke*

The cross-bridge detachment from the main force-generating state AM*D_H_ involves a complex series of events. First, a transition ([Albet-Torres et al. 2009](#_ENREF_1); [Capitanio et al. 2006](#_ENREF_5)) between the AM*D_H_ and an AMD state is required to open the nucleotide pocket for MgADP release ([Albet-Torres et al. 2009](#_ENREF_1); [Nyitrai and Geeves 2004](#_ENREF_21)), governed by rate function (cf. ([Walcott et al. 2012](#_ENREF_33))):

$k_{5}\left( x \right)=k_{5}\left( x_{1} \right)exp(G)\cdot\exp\left( \frac{{ks}\cdot\left| x \right|\cdot\delta x}{k_{B}T} \right)$ (S8)

where δx=x_2_-x_3_ (Table S1) corresponds to the difference in position for minimum free energy of the AM*D_H_ and AMD states whereas k_s_ is the stiffness of strongly bound states and δG=ΔG_AM*DH-AMD_+( ks/2)(x_2_^2^-x_3_^2^)/k_B_T (see Table S1), corresponds to the difference in free energy between the AM*D_H_ and the AMD states states at x=0 nm. Finally, k_B_ is the Bolzmann constant and T is the absolute temperature. As motivated previously ([Månsson 2010](#_ENREF_18)) the AMD, AM and AMT states are lumped together, giving the following detachment rate (k_off_(x)) for the transition from the AMD to the MT state (assuming [MgADP] = 0 mM):

k_off_(x)$=\frac{k_{2}(x)k_{6}\left[ MgATP \right]}{\frac{k_{6}}{K_{1}}+(k_{2}(x)+k_{6})\left[ MgATP \right]}=\frac{k_{2}(x)\left[ MgATP \right]}{\frac{1}{K_{1}}+\frac{k_{2}(x)}{k_{6}}\left[ MgATP \right]+[MgATP]}$ (S9)

where

$k_{2}\left( x \right)=k_{2}(0)exp \left( \frac{k_{s}\cdot\left| x \right|{\cdot x}_{crit}}{k_{B}T} \right)$ (S10)

The rate constants k_2_(0) and k_6_ govern ATP induced detachment from the AMT state at x=0 and ADP-release from the AMD state, respectively. Further, K_1_ is the equilibrium constant for MgATP binding to the AM state (Fig. 1A) and x_crit_ is a strain parameter ([Bell 1978](#_ENREF_2)) defining strain-dependence of the MgATP induced detachment. An overall detachment rate function from the AM*D_H_ state is given by:

k´_off_(x)=$\frac{k_{off}(x)k_{5}(x)}{k_{off}\left( x \right)+k_{5}(x)}$ (S11) Whereas it is possible to lump the transitions between the AMD, AM and AMT states (k_2_(x), k_6_ and K_1_) together into one transition governed by $k_{off}(x)$, accounting for [MgATP] dependence according to Eq. S9, it is of critical importance to treat the rate function k_5_(x) separately. Otherwise, key features of the cross-bridge cycle would not be possible to reproduce. For instance contractile and kinetic effects of the drug amrinone were not observed ([Albet-Torres et al. 2009](#_ENREF_1)) in the absence of strain in the myosin head (e.g. it was not seen with one-headed myosin subfragment 1 but only with two-headed HMM in solution). In order to reproduce the effect, a model with a strain-dependent transition governed by k_5_(x) was essential and all drug effects were reproduced by reduction in k_5_(x). Clearly there is the possibility that not only drugs but also mutations may act by affecting this strain-dependent transition prior to ADP-dissociation. Furthermore, the separate strain-dependent transition governed by k_5_(x) accounts for the second step in the optical tweezers studies by Captianio et al (2006) and also explains a different predominant actomyosin-ADP state during cross-bridge cycling than after ADP addition to actomyosin in solution ([Sleep and Hutton 1980](#_ENREF_27)).

Strain-dependence of the MgATP-induced detachment (k_2_(x)), in addition to strain-dependence of k_5_, seems to be required ([Persson et al. 2013](#_ENREF_24)) in a cross-bridge model with linear cross-bridge elasticity that exhibits high stiffness also in the drag-stroke region. Otherwise, it is not possible to reproduce the high sliding velocities (> 10 µm/s) at physiological temperatures. For instance, increasing the rates or strain-dependence associated with step 5 would not solve the problem because k_2_ is rate limiting for detachment at large negative x-values. On the other hand, increasing k_2_ further without assuming strain-dependence does not fit with results from solution kinetics ([Nyitrai et al. 2006](#_ENREF_22)). The issue with high velocities has not come up in previous model studies by others ([Walcott et al. 2012](#_ENREF_33)). The reason is that simulated velocities were compared to in vitro motility assay data that showed appreciably lower velocity than seen physiologically in muscle cells. Whereas the strain-dependence of k_2_(x) is not required to account for the high velocity if cross-bridge stiffness is assumed non-linear ([Persson et al. 2013](#_ENREF_24)), the latter assumption complicates the simulation procedure. Therefore, we maintained linear cross-bridge elasticity in a majority of the simulation runs and assumed an appreciable strain-dependence of k_2_(x). When non-linear cross-bridge elasticity was nevertheless introduced, this was only done in a simplified way where only the AM/AMADP state was assumed to have lower stiffness for x<0 nm.

*Contractile activation, filament compliance and target zones on actin filaments for myosin binding*

The present model assumes maximum Ca^2+^ activation. First of all, this means that all possible myosin binding sites on actin are available and no cooperative activation processes affect kinetic processes. Furthermore, all myosin heads are assumed to be free to swing out from the underlying surface without inhibiting effects related to myosin binding protein C ([Linari et al. 2015](#_ENREF_16)) or lack of phosphorylation of the myosin regulatory light chains ([Stewart et al. 2009](#_ENREF_29)). Furthermore, it is assumed (Fig. 1C) that a single site on the actin filament is within reach of one given myosin cross-bridge and that only one of the two globular units (heads) of each myosin molecule can bind simultaneously to a given target zone (here with one site only) with neighboring zones separated by 36 nm. Such a separation between target zones is broadly consistent with optical tweezers observation of myosin binding along an actin filament ([Capitanio et al. 2006](#_ENREF_5); [Steffen et al. 2001](#_ENREF_28)). Importantly, even if there are three separate binding sites associated with each target zone ([Capitanio et al. 2006](#_ENREF_5); [Steffen et al. 2001](#_ENREF_28)), the simplification of assuming only one site within reach for a given cross-bridge would not appreciably change the simulated contractile properties except for scaling effects ([Månsson 2010](#_ENREF_18)).

Effects of filament compliance ([Huxley et al. 1994](#_ENREF_13); [Wakabayashi et al. 1994](#_ENREF_32)) is not included in the simulations. This has no significance in a majority of the simulated records under steady-state conditions when force is constant. Neither is it of any importance in single molecule events when compliance of the single cross-bridge is appreciably higher than that of the filament. Finally, when filament compliance would affect the results such as during stretch (Fig. S3) or during rise of isometric tension (Fig. 4B), the effect would be small ([Månsson 2010](#_ENREF_18)).

*Actomyosin in solution*

The actomyosin ATPase in solution, V_ATPase_, vs the actin concentration can be derived from the kinetic scheme in Fig. 1A in the main paper and the kinetic constants at the minima (x_1,_ x_2_ and x_3)_ of the free-energy diagrams in Fig. 1B. It is assumed that the concentrations of inorganic phosphate and ADP are zero, phosphate release is rapid, the hydrolysis reaction (step 3) is irreversible and the AM*D_L_/AM*D_H_ equilibrium is instantaneous with a ratio

$\frac{\mathrm{AM}^{*}D_{H}}{\mathrm{AM}^{*}D_{H}+\mathrm{AM}^{*}D_{L}}$ close to 1. Then:

$\frac{1}{V_{ATPase}}=\frac{1}{k_{3+}}+\frac{1}{{K_{w}/\left[ actin \right]_{eff}k}_{a}\left( x_{1} \right)[actin]}+\frac{1}{k_{+P}(x_{1})}+\frac{1}{k_{5}(x_{2})}+\frac{1}{k_{off}(x_{1})}$ (S12)

where $\left[ actin \right]_{eff}$is the effective actin concentration in muscle and [actin] is the actin concentration (on monomer basis) in solution. If we now set ${k_{+P}\equiv k}_{+P}(x_{1})$, ${k_{5}\equiv k}_{5}(x_{2})$ and ${k_{off}\equiv k}_{off}(x_{1})$ then:

$V_{ATPase}=\frac{V_{max}[actin]}{K_{M}+[actin]}$ (S13)

with

$V_{max}=\frac{k_{+P}}{1+\frac{k_{+P}}{k_{3+}}+\frac{k_{+P}}{k_{5}}+\frac{k_{+P}}{k_{off}}}$ (S14)

and

$K_{M}=\frac{1}{{(K}_{w}/\left[ actin \right]_{eff})(1+\frac{k_{+P}}{k_{3+}}+\frac{k_{+P}}{k_{5}}+\frac{k_{+P}}{k_{off}})}$ (S15)

*Comments on parameter values*

All strongly bound myosin heads were assumed to exhibit a stiffness of 2.8 pN/nm as further motivated in Table S1. The stiffness of weakly bound myosin heads was taken as 0.02 pN/nm in the simulations with free-energy minimum at x=7.7 nm. The basis for these parameter values is evidence for negligible resistive effect of the weak-binding state on sliding velocity in muscle cells and in recent in vitro motility assay studies at close to physiological ionic strength ([Gulati and Podolsky 1981](#_ENREF_10); [Vikhorev et al. 2008](#_ENREF_36)). Furthermore, one would expect a low stiffness with the myosin head “rolling” around a weak electrostatic attachment point on the thin filament. The characteristics of the strain-dependent transition between the AM*D_H_ and an AMD state as well as the subsequent actomyosin dissociation steps are consistent with recent findings ([Albet-Torres et al. 2009](#_ENREF_1); [Capitanio et al. 2006](#_ENREF_5); [Persson et al. 2013](#_ENREF_24)).

The model parameters were directly obtained from experimental data in the literature except the value of k_5_(x_2_)=k_5_*exp($G$). This parameter was adjusted, with starting point in values from recent work ([Albet-Torres et al. 2009](#_ENREF_1)), to give an approximate fit of the model to the force-velocity-relationship. The parameter K_w_ was also varied to test its effect on several model predictions. With k_2_ and k_6_ values from the litterature, the rate function k_5_(x) is a key determinant both of the maximum velocity of shortening and the shape of the force-velocity relationship ([Albet-Torres et al. 2009](#_ENREF_1)). In the process of fitting the model to the latter relationship, experimentally constrained parameter values were varied within their experimentally observed ranges ( <± 20 % of mean) together with up to ten-fold variation in k_5_(x2)exp($G$). The fitting was deemed satisfactory when the shape of the force-velocity-relationship was faithfully reproduced (e.g. correct type of non-hyperbolic deviation at high loads) and when the maximum velocity differed from experimental values by <± 20 %. No detailed quantitative fitting (e.g. based on minimization of mean squared residuals) was judged as meaningful because: 1. any model merely approximates complex biological behavior and 2. experimental data against which the model is validated have been obtained from different labs and, inevitably, to some extent varying experimental conditions.

Model parameter values were constrained by litterature data for fast mammalian skeletal muscle fibers of two-headed myosin motor fragments at 25-30 ^o^C and ionic strengths of 130-200 mM whenever possible. This is close both to physiological conditions and typical conditions for experiments using skinned muscle fibers or in vitro motility assays. Exceptions from the pre-selected constraints for the parameter values are specified in Tables S1-S2 and motivated in the footnotes of Tables S1-S2.

Whereas strain-dependence of equilibrium constants are defined by litterature data via the free-energy diagrams, individual rate constants are not. In the present implementation of the model this introduces ambiguities in the exact strain dependence of the rate function for strong attachment preceeding Pi-release and the reversal of this reaction. Naturally, no ambiguities are introduced into transitions that are, anyway, treated as rapid equilibria.

In most cases, similar stiffness is assumed for positive and negative x. However, in some simulations the elasticity was assumed to be non-linear with lower stiffness at negative x. The aim was to test what general type of changes (rather than detailed effects) such a modification of the model would lead to. Therefore we took the simplified approach to assume that cross-bridge stiffness in the AM-state (also AMD and AMT states) was decreased step-wise, about 10-fold at x=0 nm, from 2.8 pN/nm at x≥ 0 nm to 0.25 pN/nm for x<0 nm. This differs from the experimental findings ([Kaya and Higuchi 2010](#_ENREF_15)) in that the latter showed a gradual rather than step-wise decrease in stiffness when going into negative x-regions and that the minimum stiffness observed for large negative x-values was lower (0.02 pN/nm). The stiffness value used here, is similar to the average stiffness in the range [-5, 0] nm in ([Kaya and Higuchi 2010](#_ENREF_15)) (their Fig. 1). As mentioned above, the simplifications are in line with the goal of the simulations. Furthermore, they can be justified because the experimental stiffness-strain relationship ([Kaya and Higuchi 2010](#_ENREF_15)) was obtained using isolated myosin filaments. The characteristics of the cross-bridge elasticity in a muscle cell or in vitro motility assay, conditions simulated here, is not known in detail.

**Supplementary Methods**

*Implementation for large ensembles by solution of differential equations*

For large ensembles steady-state conditions with constant velocity, v, were simulated. In these cases the model was implemented by solving differential equations for the state probabilities in the generic form:

$\frac{da_{j}}{dx}=(\sum_{i}^{n1} k_{ij}\left( x \right)a_{i}\left( x \right)-\sum_{k}^{n2} k_{jk}{(x)a}_{j(x)})/v$ (S16)

where a_i_(x) and a_j_(x) are the state probabilities for the MT, MDP, AMDP, states etc. in Fig. 1 and the rate functions k_ij_(x) and k_jk_(x) represent rates of transitions into n1 neighboring state and out of state a_j_ (into n2 other states), respectively. The model simulations were implemented by numeric solution of the master equations (Eq. S16) followed by calculation of observable parameters (force and ATP turnover rate) from appropriate state probabilities ([Månsson 2010](#_ENREF_18)) by averaging over the inter-site distance (36 nm) along the actin filament. For instance, average force *<F>* (in pN) per myosin head (whether attached to actin or not) is calculated as:

$<F>= \frac{\sum_{1}^{n1} \int_{-18}^{18} {{ks}_{i}}\left( x \right)a_{i}\left( x \right)(x-x_{i})dx}{\sum_{i}^{n1} \int_{-18}^{18} a_{i}\left( x \right)dx}$ (S17)

where ks_i_ is cross-bridge stiffness (pN/nm) for the state with probability governed by a_i_(x), and x_i_ is the x-value with free energy minimum for this state. Stiffness of detached states is zero, x is in nm and summing is over all n1 states. The rapid equilibria (between the MADP and AMDP states and between the AMD_L_ and AMD_H_ states) were simulated using high numerical values of the forward k_ij_ and backward k_ji_ rate functions. In the large ensemble models there is no competition between myosin heads for binding to a given actin filament site. This follows from the simplifying assumption that only one head in the myosin head pair is capable of binding at a time and that only one head pair is within reach of a given binding site on the thin filament site. The latter follows from the arrangement of the myosin heads on the thick filament in the sarcomere and our assumption of only one site every 36 nm along the actin filament.

*Implementation for small ensembles and single molecules using Monte-Carlo simulations*

In order to simulate in vitro motility assay results, we made the simplifying assumption that HMM motor fragments are adsorbed to the motility assay surfaces with uniform density, ρ and that myosin heads in a band of d=30 nm width around the long axis of the filament, are available for binding. Then we assumed that all 36 nm intervals along an actin filament, with the myosin binding site in the center, are identical, with the total number of available myosin heads (n=ρdl) along the entire filament of length, l, distributed as uniformly as possible between 360 bins each 0.1 nm wide. The number of heads was not exactly identical for all bins because the total number was rarely divisible by 360. Therefore, we used an algorithm that gave as even distribution as possible with a maximum difference of one myosin head between bins. A doubling of the number of bins per 36 nm did not noticeably modify the simulation results.

All myosin heads were initially assumed to be in the MDP state. The time interval, Δt, until the first update event in the simulations was calculated using the Gillespie algorithm ([Gillespie 1976](#_ENREF_9)) from the inverse rate summed over all possible chemical transitions at each value of x=x_bin_:

$\Delta t=\frac{1}{\sum_{i=1}^{ntot} \sum_{xbin=1}^{360} {\sum_{j}^{ntrans} k_{ij}(xbin)a}_{i}(xbin)}$ (S18)

where i represents the chemical states in Fig. 1A, except the states in rapid equilibrium. Now, a_i_(xbin) represents the number of myosin heads in each of the chemical states for each given value of xbin. Finally, for all biochemical states i, and all xbin-values, a number (ntrans) of chemical transitions, out of the state i, are possible each with rate constant k_ij_(xbin). At time, Δt, the transition to occur as well as the xbin value, were selected by comparing the probabilities of all possible transitions (and x_bin_-values) to a random number in the range [0, 1] from a uniform probability distribution.

After updating with a chemical event (hydrolysis, attachment, detachment etc.), the average strain of the myosin head was adjusted, either 1. to exactly balance an external load in the simulation of a load-clamp experiment, 2. to reproduce a constant velocity sliding/shortening/stretch in a velocity clamp experiments or 3. not at all in order to simulate isometric contraction. A special case of the load-clamp is simulation of an unloaded (standard) in vitro motility assay or unloaded shortening velocity in a muscle cell in which case the constant load is zero.

Following the adjustments of the cross-bridge distribution to achieve force balance, the rapid equilibria governed by K_W_(x) and K_LH_(x) were updated by assuming that the number of heads in the two possible states at each x_bin_-value obeys a binomial distribution (Bin (n, p)) where n is the total number of myosin heads in the two states in equilibrium for the given bin and p is the probability that the state most clockwise in Fig. 1A is populated. Thus, for the equilibrium governed by K_W_(x), the probability that the AMDP state is populated is given by p= K_W_(xbin)/ (K_W_(xbin)+1)).

Subsequently, a new Gillespie step followed with calculation of the time to the next event, selection of this event, update of cross-bridge strains etc. as described above. In the simulation of in vitro motility assay data (in contrast to muscle data; see above) it is important to account for the fact that a large number of myosin heads may be within reach of a given actin site whereas only one head can bind to each given site. The low number of actin sites (Filament length (µm)/0.036 nm, i.e.27.8 µm^-1^) compared to the number of available myosin heads (150 µm^-1^) is taken into account by making the probability of myosin head attachment to actin proportional both to the total number of myosin heads in the MDP and AMDP states and to the number of free actin filament sites. Furthermore, we labelled the actin filament sites along a filament and randomly assigned a given attachment event to previously unoccupied sites. Simulation of the inclusion of rigor-like “dead” cross-bridges (in an AM^D^-state; see main paper) was achieved by first uniformly, in proportion to their number-fraction, distribute these heads between the x-bins. We used a detachment rate function according to Nishizaka ([Nishizaka et al. 2000](#_ENREF_20)):

$k_{rigor}\left( x \right))$=$k_{rigor}\left( 0 \right)\exp\left( \frac{k_{s}\cdot\left| x \right|{\cdot x}_{rigor}}{k_{B}T} \right)$ (S19)

where k_rigor_(0) = 0.016 s^-1­^ and x_rigor_= 2.7 nm.

We used a high attachment rate function for attachment to the available actin site in general agreement with the free-energy diagram for the AM state in Fig. 1:

k_on-rigor_(x)≈k_+P_(x)=kon0_rigor_ exp[$\Delta$G_AM-rigor_/2 - (ks/2)(x-x_3_)^2^/k_B_T] (S20)

where kon0_rigor_=10 s^-1^ and$\Delta$G_AM-rigor_=18. Because the attachment rate is very high compared to the detachment rate the simulation results were not altered by orders of magnitude changes of kon0_rigor_.

In each step of the Gillespie algorithm, attachment or detachment of rigor bridges was selected in accordance with the probability of these events relative to other events. The Monte-Carlo simulation procedure for small ensembles as described above was modified to accommodate simulation of single-molecule behavior. Essentially, the waiting time between events was calculated as in the Gillespie algorithm and then applied to the only possible transition followed by instantaneous adjustment of force to externally imposed load.

*Comparison of simulated data for small and large ensembles*

A range of phenomena were simulated using the implementation approaches for both small and large ensembles. In order to achieve a fair comparison, the model for small ensembles was implemented for as large ensembles as practically possible by simulating the results for a filament length of 20 µm using the highest reasonable myosin head density of 5000 µm^-2^. Using a higher surface density would noticeably reduce isometric force per head relative to that in the large-ensemble model due to competition between several myosin heads for a given actin site. For comparison of the two simulation methods, all parameters values were identical. However, whereas finite values of the backward and forward rates associated with Kw and Keq was used in the differential equation based ensemble simulations these rates were infinite in the Monte-Carlo approach, i.e. we assumed that the equilibrium was attained instantaneously. These assumptions account for a slightly higher maximum sliding velocity using Monte-Carlo simulations. The slightly lower maximum force in Monte-Carlo simulations than in the differential equation based implementation may also be partly accounted for by this difference in computational treatment of rapid equilibria. Furthermore, the force is reduced in the Monte-Carlo simulations due to some competition between myosin heads for a given actin filament site (see above).

**Table S1**. Parameter values^a^ determining shape of free energy diagrams for simulation of contractile properties of fast mammalian muscle at 25-30 ^o^C

| **Parameter** | **Numerical value used** | **Range from litterature** | **References** | **Comment** |
| --- | --- | --- | --- | --- |
| x_w_ (AMDP) | 7.7 nm | Set to be equal to x_1_ |  | Not critical parameter value; low stiffness of AMDP states |
| x_1_ (AM*D_L_) | 7.7 nm | ~8 nm | ([Kaya and Higuchi 2010](#_ENREF_15))^b^ | Rabbit full length myosin in filaments, <20 mM IS; 20 ^o^C |
| x_2_ (AM*D_H_) | 1.0 nm | 0.9-1.1 nm | ([Capitanio et al. 2006](#_ENREF_5))^c^ | Myosin subfragment 1, fast mouse muscle; 22 ^o^C; IS < 30-50 mM |
| x_3_ | 0 nm |  | See text | By definition |
| $\Delta$G_w_  (MDP- AMDP) | Varied between 0 and 2.5 k_B_T i.e. K_w_: 1-12 | ~0 k_B_T | ([Brenner et al. 1986](#_ENREF_4)) | Free energy difference between the MDP state and the AMDP state; $\Delta$G_w_ ≈ 0 shown in Fig. 1B |
| $\Delta$G_AMDP-AM*DL_ (AMDP –AM*D_L_) | 0.7 k_B_T -ln([P_i_]/K_C_) + elastic contribution | K_C_ below. Strain dep free energy to account for rate of force generation in Pi-transients | From ([Dantzig et al. 1992](#_ENREF_7)) and  Q_10_ = 2.7-3.7 in range 20-35^o^C ([Brenner and Eisenberg 1986](#_ENREF_3); | Difference between the free energy minima of the AMDP and the AM*D_L_ states |
| $\Delta$G_AM*DL-_  _AM*DH_  (AM*D_L_- AM*D_H_) | 15 k_B_T | $\Delta$G_AM*DL-_  _AM*DH_  _+_  $\Delta$G_AM*DH-_  _AMD_  (10-20 k_B_T) | ([Karatzaferi et al. 2004](#_ENREF_14); [Kaya and Higuchi 2010](#_ENREF_15)) | Defined as above. Consistent with cross-bridge stiffness and parameter values x_1_ and x_2._ Rabbit myosin filaments (see above) and rabbit muscle fibers ([Karatzaferi et al. 2004](#_ENREF_14)) |
| $\Delta$G_AM*DH-_  _AMD_  (AM*D_H_ AMD) | 2 k_B_T | $\Delta$G_AM*DL-_  _AM*DH +_  $\Delta$G_AM*DH-_  _AMD_  (10-20 k_B_T)  1-2 k_B_T | ([Kaya and Higuchi 2010](#_ENREF_15); [Persson et al. 2013](#_ENREF_24)) | Used in previous work to fit effects of drug amrinone and force-velocity data. |
| $\Delta G_{ATP}$ | 13.1 + ln ([MgATP]/ ([MgADP][Pi]) k_B_T | Free energy of ATP-hydrolysis | ([Pate and Cooke 1989](#_ENREF_23)) |  |
| ks | 2.8 pN/nm | 2.5-2.8 pN/nm | ([Kaya and Higuchi 2010](#_ENREF_15)) ^b^ |  |
| ksw | 0.02 pN/nm | - |  | Not allowed to affect velocity |

Footnotes to Table S1

NA: Not applicable; IS: ionic strength

^a^ The parameter values were from two-headed myosin motor fragments from fast skeletal muscle of rabbit at 25-30^o^C, ionic strength 130-200 mM, pH 7-8 unless otherwise stated.

^b^Selected despite low ionic strength because other experiments use subfragment 1 or are believed to exhibit appreciable series compliance. Furthermore, the results are similar to those obtained under near physiological conditions of 200 mM ionic strength and physiological interfilament distances ([Linari et al. 2007](#_ENREF_17)) in skinned rabbit psoas muscle fibers, suggesting temperature independent cross-bridge stiffness of 1.7 pN/nm and average cross-bridge strain of 3.9 nm during isometric contraction at 20 ^o^C. This may be compared to 2.5 nm for the present data (assuming x_1_=7.7 nm) from dividing average isometric force with the stiffness due to attached cross-bridges (calculated but not shown). In addition Linari et al. ([Linari et al. 2007](#_ENREF_17)) discussed the possibility that the lower stiffness of rabbit psoas actomyosin cross-bridges than frog cross-bridges (3.3 pN/nm; ([Piazzesi et al. 2007](#_ENREF_25))) might be partly attributed to the use of skinned rather than intact fibers for the rabbit data. All this motivates the current estimates of x_1_ and k_s_ from the low ionic strength rabbit psoas data in ([Kaya and Higuchi 2010](#_ENREF_15)).

^c^Consistent with fits of force-velocity data of muscle cells ([Albet-Torres et al. 2009](#_ENREF_1); [Månsson 2010](#_ENREF_18)) including effects of the drug amrinone. The value of x_2_ is also about half that (2 nm) found by Veigel et al. ([Veigel et al. 2003](#_ENREF_34)) for one-headed smooth muscle myosin fragments under similar experimental conditions. This accords with a second step length shorter than 1.8 nm for fast rabbit psoas considering the structural data where a 3.5 nm length step was associated with ADP release in smooth muscle myosin ([Whittaker et al. 1995](#_ENREF_35)).

**Table S2**. Parameter values^a^ defining rate functions and kinetic constants for simulation of contractile properties of fast mammalian muscle at 25-30 ^o^C.

| **Parameter** | **Numerical value used** | **Litterature value(s)** | **References** | **Comment** |
| --- | --- | --- | --- | --- |
| k_+3_ + k_-3_  (Recovery stroke+hydrolysis) | 220 s^-1^ | 200-500 s^-1^ Assuming Q_10_ in range 3-4 ([Woledge et al. 1985](#_ENREF_37)) | ([Sleep et al. 2005](#_ENREF_26); [Woledge et al. 1985](#_ENREF_37)) and references therein |  |
| K_3_ | 10 | 2-10 | ([Sleep et al. 2005](#_ENREF_26); [Woledge et al. 1985](#_ENREF_37)) and references therein | From myosin subfragment 1 from fast muscle |
| K_LH_(x) |  |  |  | Follows from free energy curves defined by parameter values above |
| k_-5_ | 2000 s^-1^ |  | From fit^b^ |  |
| Kc | 10 mM | ~1-10 mM (temp corrected) | ([Dantzig et al. 1992](#_ENREF_7)) | From fast skinned muscle fiber phosphate transients, from data at 20-25 ^o^C |
| k_b0_ | 100 s^-1^ | ~100 s^-1^ | ([Dantzig et al. 1992](#_ENREF_7)) | From fast skinned muscle fiber phosphate transients, from data at 20-25 ^o^C |
| x_crit_ | 0.6 nm | < 0.2 nm (see further, Theory) | ([Capitanio et al. 2012](#_ENREF_6)) | From myosin subfragment 1 of fast mouse muscle at 20 ^o^C and ionic strength < 50 mM |
| k_6_ | 5000 s^-1^ | >3500 s^-1^ | ([Nyitrai et al. 2006](#_ENREF_22)) | Fast rabbit myosin subfragment 1 |
| k_-6_ | 14 290 mM^-1^ s^-1^ | >10 000 mM^-1^ s^-1^ | ([Nyitrai et al. 2006](#_ENREF_22)) | Diffusion limited^c^ |
| Physio-logical [Pi] | 0.5 mM | ~ 0.5 mM | ([Debold et al. 2011](#_ENREF_8)) |  |
| K_1_ | 1.7 mM^-1^ | 1.7 mM^-1^ | ([Nyitrai et al. 2006](#_ENREF_22)) | From fast rabbit myosin subfragment 1 |
| k_2_ | 1400 s^-1^ | 1400 s^-1^ | ([Nyitrai et al. 2006](#_ENREF_22)) | From fast rabbit myosin subfragment 1; Temperature corrected from 25 ^o^C data in Fig.6 of ([Nyitrai et al. 2006](#_ENREF_22)). |

Footnotes to Table S2

^a^ The parameter values were from two-headed myosin motor fragments from fast skeletal muscle of rabbit at 25-30^o^C, ionic strength 130-200 mM, pH 7-8 unless otherwise stated.

^b^Fit of force velocity relationship. See text.

^c^From the assumption of a diffusion limited rate constant > 10 000 mM^-1^ s^-1^ (14 000 mM^-1^ s^-1^)

**Fig. S1. Comparison of simulations based on solution of differential equations in state probabilities (full circles and lines) and simulations for as large ensembels as possible using Monte-Carlo approach (open squares). A.** Force-velocity relationship for physiological conditions with force per total number of available heads (attached and unattached). Inset. Force-velocity relationship normalized to maximum force and maximum velocity. **B**: Simulated force records (noisy records, left vertical axis) in response to isovelocity shortening ramps (straight lines, right axis) at different velocities starting at time zero. **C.** Velocity plotted against [MgATP]. Inset: Data normalized to velocity at 5 mM MgATP. **D.** Maximum isometric force (squares and circles) and maximum velocity (triangles) as function of the concentration of inorganic phosphate. Full and dashed purple line: hyperbolic curves fitted to experimental data from ([Tesi et al. 2002](#_ENREF_31)) at ionic strength of 200 mM (15-20 ^o^C), for fast and slow rabbit skeletal muscle, respectively.

**Fig. S2. Comparison of simulated data (black; linear compliance; standard parameter values [Tables S1-S2]) and experimental data (purple). A.** Effects of load on single molecule ATP-dependent detachment rate at 10 (circles) and 50 µM (squares) [MgATP]. Experimental data of Capitanio et. al. (([Capitanio et al. 2012](#_ENREF_6))) measured from their Figure 4d. **B.** MgATP-dependent detachment rate at near saturating [MgATP]. Simulated data for rabbit fast myosin at 30 ^o^C compared to experimental data at >2 mM [MgATP] from cardiac β-myosin subfragment 1 at 23 ^o^C ([Sung et al. 2015](#_ENREF_30)) derived by fitting the Bell-Evans ([Bell 1978](#_ENREF_2)) model (rate=k0exp(-Fδ1/k_B_T)) to the data where F is force and δ1 is a critical strain. The experimentally derived equation with δ1=0.8±0.1 nm is here scaled by increasing k0 (87±7 s^-1^ in ([Sung et al. 2015](#_ENREF_30))) to our single molecule detachment rate at zero load. Isometric relaxation rate (from ([Månsson et al. 1989](#_ENREF_19)); 10 s^-1^) and isometric ATPase rate per attached head from fast mammalian skeletal muscle fibers at 30 ^o^C ([Hilber et al. 2001](#_ENREF_11)) are below purple line line (100 s^-1^). This seems to indicate lower ATP-dependent detachment rate at these loads for actomyosin from fast rabbit muscle than inferred from the experiments in A-B, using myosin subfragment. Generally, differences between model and experimental data may be attributed to: 1. Different motor fragments (subfragment 1), temperature, myosin isoform and species in the experiments than assumed in the simulations (two-headed myosin at 30 ^o^C, fast skeletal muscle isoform of rabbit), 2. the assumption of appreciable strain-dependence of k_2_(x) that was required in our model to account for high velocity to avoid the assumption of non-linear filament compliance.

**Fig. S3. Velocity vs filament length in the in vitro motility assay. A.** Simulated velocity vs filament length (5000 active myosin heads per µm^2^) for standard conditions assuming K_w_=12 (open symbols, full lines) and K_w_=1 (crosses and dashed line, stars and dotted lines) compared to experimental in vitro motility assay data (filled symbols ([Persson et al. 2013](#_ENREF_24))). Simulated and experimental data at 1mM (black), 0.1 mM (red) and 0.01 mM (blue) [MgATP]. Filled lines represent fits of Eq. 1 to the simulated data for K_w_=12 and 1 independent myosin binding site are assumed per 36 nm of the actin filament (n_s_=1). Dashed line and dotted lines (K_w_=1) represent fits of Eq. 1 to simulation results with n_s_=1 and n_s_=3, respectively. **B.** Fraction of attached myosin heads in strongly bound states AM*D_L_, AM*D_H_, AMD, AM and AMT from simulations plotted against duty ratio from fit of Eq. 1 to the simulated data. The filled symbols and full line show data that are also illustrated in Fig. 3 (main paper; K_w_=12, n_s_=1). The stars and dotted line represent data in Fig. S3A (K_w_=1, n_s_=3). Horizontal error bars are the 95 % CIs obtained in the non-linear regression fit of Eq. 1 with maximum velocity and duty ratio as varying parameters in the regression analysis. Dashed straight line has slope 1.

**B**

**A**

1

2

3

4

**Time (s)**

**Fig. S4. “Eccentric contraction” of single actin filament. A**. Simulated isometric force development with superimposed stretch (after 0.025 s) of a filament of length 20 µm under otherwise standard conditions. Green trace: Force-dependent detachment during stretch with rate function similar to that for strain-dependent detachment of rigor cross-bridges found in ([Nishizaka et al. 2000](#_ENREF_20)). In this case rapid reattachment to neighboring site was not assumed. Black trace: Same detachment rate function during stretch as for green trace but immediate reattachment to neighboring site along actin filament (slippage) **B.** Schematic illustration of “slippage” during stretch (towards right; arrow) of actin filament with rapid detachment and reattachment of cross-bridges: 1. The cross-bridge is stretched with strain in lever arm resulting in 2. rapid detachment followed by 3. instantaneous reattachment to neighboring site and 4. attachment of new myosin heads to initial site.

**Supplementary References**

Albet-Torres N et al. (2009) Drug effect unveils inter-head cooperativity and strain-dependent ADP release in fast skeletal actomyosin J Biol Chem 284:22926–22937

Bell GI (1978) Models for the specific adhesion of cells to cells Science 200:618-627.

Brenner B, Eisenberg E (1986) Rate of force generation in muscle: correlation with actomyosin ATPase activity in solution Proc Natl Acad Sci U S A 83:3542-3546

Brenner B, Yu LC, Greene LE, Eisenberg E, Schoenberg M (1986) Ca2+-sensitive cross-bridge dissociation in the presence of magnesium pyrophosphate in skinned rabbit psoas fibers Biophys J 50:1101-1108 Capitanio M et al. (2006) Two independent mechanical events in the interaction cycle of skeletal muscle myosin with actin Proc Natl Acad Sci U S A 103:87-92

Capitanio M et al. (2012) Ultrafast force-clamp spectroscopy of single molecules reveals load dependence of myosin working stroke Nature Methods 9:1013-1019 doi:10.1038/nmeth.2152

Dantzig JA, Goldman YE, Millar NC, Lacktis J, Homsher E (1992) Reversal of the cross-bridge force-generating transition by photogeneration of phosphate in rabbit psoas muscle fibres J Physiol 451:247-278

Debold EP, Turner MA, Stout JC, Walcott S (2011) Phosphate enhances myosin-powered actin filament velocity under acidic conditions in a motility assay Am J Physiol Regul Integr Comp Physiol 300:R1401-1408

Gillespie DT (1976) A general method for numerically simulating the stochastic time evolution of coupled chemical reactions J Comp Phys 22:403-434

Gulati J, Podolsky RJ (1981) Isotonic contraction of skinned muscle fibers on a slow time base: effects of ionic strength and calcium J Gen Physiol 78:233-257

Hilber K, Sun YB, Irving M (2001) Effects of sarcomere length and temperature on the rate of ATP utilisation by rabbit psoas muscle fibres J Physiol 531:771-780

Huxley AF, Simmons RM (1971) Proposed mechanism of force generation in striated muscle Nature 233:533-538

Huxley HE, Stewart A, Sosa H, Irving T (1994) X-ray diffraction measurements of the extensibility of actin and myosin filaments in contracting muscle Biophys J 67:2411-2421

Karatzaferi C, Chinn MK, Cooke R (2004) The force exerted by a muscle cross-bridge depends directly on the strength of the actomyosin bond Biophys J 87:2532-2544

Kaya M, Higuchi H (2010) Nonlinear elasticity and an 8-nm working stroke of single myosin molecules in myofilaments Science 329:686-689

Linari M et al. (2015) Force generation by skeletal muscle is controlled by mechanosensing in myosin filaments Nature 528:276-279

Linari M, Caremani M, Piperio C, Brandt P, Lombardi V (2007) Stiffness and fraction of Myosin motors responsible for active force in permeabilized muscle fibers from rabbit psoas Biophys J 92:2476-2490

Månsson A (2010) Actomyosin-ADP states, inter-head cooperativity and the force-velocity relation of skeletal muscle Biophys J 98:1237-1246

Månsson A, Morner J, Edman KA (1989) Effects of amrinone on twitch, tetanus and shortening kinetics in mammalian skeletal muscle Acta Physiol Scand 136:37-45

Nishizaka T, Seo R, Tadakuma H, Kinosita K, Jr., Ishiwata S (2000) Characterization of single actomyosin rigor bonds: load dependence of lifetime and mechanical properties Biophys J 79:962-974

Nyitrai M, Geeves MA (2004) Adenosine diphosphate and strain sensitivity in myosin motors Philos Trans R Soc Lond B Biol Sci 359:1867-1877

Nyitrai M, Rossi R, Adamek N, Pellegrino MA, Bottinelli R, Geeves MA (2006) What limits the velocity of fast-skeletal muscle contraction in mammals? J Mol Biol 355:432-442

Pate E, Cooke R (1989) A model of crossbridge action: the effects of ATP, ADP and Pi J Muscle Res Cell Motil 10:181-196.

Persson M, Bengtsson E, ten Siethoff L, Månsson A (2013) Nonlinear cross-bridge elasticity and post-power-stroke events in fast skeletal muscle actomyosin Biophys J 105:1871-1881

Piazzesi G et al. (2007) Skeletal muscle performance determined by modulation of number of Myosin motors rather than motor force or stroke size Cell 131:784-795

Sleep J, Irving M, Burton K (2005) The ATP hydrolysis and phosphate release steps control the time course of force development in rabbit skeletal muscle J Physiol 563:671-687

Sleep JA, Hutton RL (1980) Exchange between inorganic phosphate and adenosine 5'-triphosphate in the medium by actomyosin subfragment 1 Biochemistry 19:1276-1283

Steffen W, Smith D, Simmons R, Sleep J (2001) Mapping the actin filament with myosin Proc Natl Acad Sci U S A 98:14949-14954

Stewart M, Franks-Skiba K, Cooke R (2009) Myosin regulatory light chain phosphorylation inhibits shortening velocities of skeletal muscle fibers in the presence of the myosin inhibitor blebbistatin J Muscle Res Cell Motil 30:17-27

Sung J et al. (2015) Harmonic force spectroscopy measures load-dependent kinetics of individual human beta-cardiac myosin molecules Nature Commun 6:7931

Tesi C, Colomo F, Piroddi N, Poggesi C (2002) Characterization of the cross-bridge force-generating step using inorganic phosphate and BDM in myofibrils from rabbit skeletal muscles J Physiol 541:187-199

Wakabayashi K, Sugimoto Y, Tanaka H, Ueno Y, Takezawa Y, Amemiya Y (1994) X-ray diffraction evidence for the extensibility of actin and myosin filaments during muscle contraction Biophys J 67:2422-2435

Walcott S, Warshaw DM, Debold EP (2012) Mechanical coupling between myosin molecules causes differences between ensemble and single-molecule measurements Biophys J 103:501-510

Veigel C, Molloy JE, Schmitz S, Kendrick-Jones J (2003) Load-dependent kinetics of force production by smooth muscle myosin measured with optical tweezers Nature Cell Biol 5:980-986

Whittaker M, Wilson-Kubalek EM, Smith JE, Faust L, Milligan RA, Sweeney HL (1995) A 35-A movement of smooth muscle myosin on ADP release Nature 378:748-751

Vikhorev PG, Vikhoreva NN, Månsson A (2008) Bending flexibility of actin filaments during motor-induced sliding Biophys J 95:5809-5819

Woledge RC, Curtin NA, Homsher E (1985) Energetic aspects of muscle contraction. Monographs of the physiological society No. 41. Academic Press, London
